# Therapeutic Targeting Strategies for Early- to Late-Staged Alzheimer’s Disease

**DOI:** 10.3390/ijms21249591

**Published:** 2020-12-16

**Authors:** You Jung Kang, Yen N. Diep, Minh Tran, Hansang Cho

**Affiliations:** 1 Department of Mechanical Engineering and Engineering Science, Center for Biomedical Engineering and Science, University of North Carolina, Charlotte, NC 28223, USA; ykang15@uncc.edu; 2 Department of Biological Sciences, Center for Biomedical Engineering and Science, University of North Carolina, Charlotte, NC 28223, USA; 3 Institute of Quantum Biophysics, Department of Biophysics, Sungkyunkwan University, 2066 Seobu-ro, Jangan-gu, Suwon-si, Gyeonggi-do 16419, Korea; yendiep@g.skku.edu (Y.N.D.); minhtran@g.skku.edu (M.T.); 4 Department of Intelligent Precision Healthcare Convergence, Sungkyunkwan University, 2066 Seobu-ro, Jangan-gu, Suwon-si, Gyeonggi-do 16419, Korea

**Keywords:** Alzheimer’s disease, therapeutic strategies, mild to severe, early to late stages, dementia, pathology

## Abstract

Alzheimer’s disease (AD) is the most common cause of dementia, typically showing progressive neurodegeneration in aging brains. The key signatures of the AD progression are the deposition of amyloid-beta (Aβ) peptides, the formation of tau tangles, and the induction of detrimental neuroinflammation leading to neuronal loss. However, conventional pharmacotherapeutic options are merely relying on the alleviation of symptoms that are limited to mild to moderate AD patients. Moreover, some of these medicines discontinued to use due to either the insignificant effectiveness in improving the cognitive impairment or the adverse side effects worsening essential bodily functions. One of the reasons for the failure is the lack of knowledge on the underlying mechanisms that can accurately explain the major causes of the AD progression correlating to the severity of AD. Therefore, there is an urgent need for the better understanding of AD pathogenesis and the development of the disease-modifying treatments, particularly for severe and late-onset AD, which have not been covered thoroughly. Here, we review the underlying mechanisms of AD progression, which have been employed for the currently established therapeutic strategies. We believe this will further spur the discovery of a novel disease-modifying treatment for mild to severe, as well as early- to late-onset, AD.

## 1. Introduction

**Figure 1:**
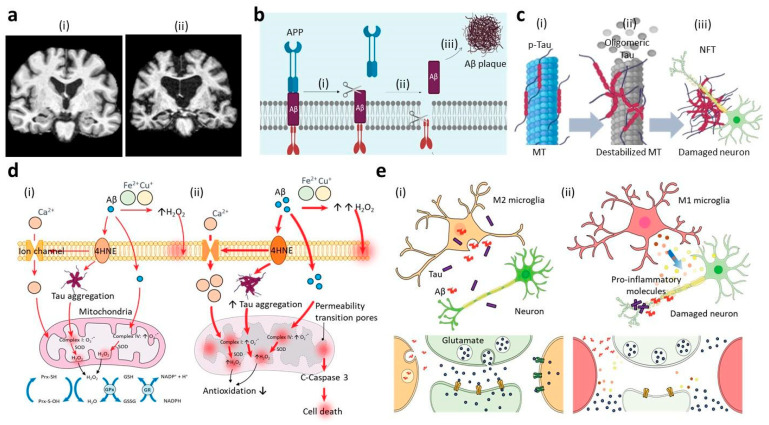
Summary of major signatures during Alzheimer’s disease (AD) progression. (**a**) Comparison of brain coronal sections from (i) healthy individual and (ii) AD patient by magnetic resonance imaging (MRI), confirming the significant damage in the AD patient having the reduced brain mass. Reproduced with permission [4]. Copyright 2018, Scientific Reports. (**b**) Amyloid-beta hypothesis: In early AD, the enzymatic cleavages of amyloid-beta precursor protein (APP) by (i) β-secretase followed by (ii) γ-secretase are dominant and result in the formation of Aβ peptides, which are hydrophobic and prone to form aggregations. (iii) In the early to intermediate stages of AD, the aggregates form into Aβ amyloid fibrils and plaques that further cause phosphorylation of tau, neuronal death, cell loss, and dementia, sequentially [81,84]. (**c**) Tauopathy: (i) Upon the hyperphosphorylation on the multiple sites of tau, the tau proteins are not able to bind to the microtubules (MT) resulting in the disruption of microtubule structures inside neuronal cells. (ii) They further form oligomeric tau, paired helical filament (PHF), and neurofibrillary tangle (NFT), consequently, and (iii) the accumulation of NTFs in the neurons increases the synaptic impairment and the neuronal death in the middle stage of AD [87,88]. (**d**) Oxidative stress: (i) Under normal or mild AD conditions, antioxidation mechanisms (e.g., mitochondrial redox cycles) can reduce the oxidative stress caused by Aβ and tau aggregations. (ii) In later stages of AD, however, the accumulation of Aβ and tau triggers the excessive production of oxidative stress and reduces the antioxidation mechanism of mitochondria or antioxidant enzymes, which increases the neuronal death [89,90]. (**e**) Neuroinflammation: (i) In the early stages, innate immune cells obtain phenotypes (e.g., M2 microglia, A2 astrocyte, etc.) serving neuroprotective roles, such as the removal of Aβ and tau aggregations and the production of anti-inflammatory cytokines, as well as neurotrophic factors. (ii) In the later AD stages, on the other hand, the population of proinflammatory immune cells (e.g., M1 microglia, A1 astrocyte, etc.) becomes dominant and increases the risk of AD by producing several neurotoxic mediators, such as oxidative sources and proinflammatory cytokines/chemokines [91,92].

Alzheimer’s disease is a worldwide public health concern as it is the most common cause of dementia, occasionally found in elderly [1,2]. Recent reports showed that nearly 50 million people were suffering from AD in the world in 2018 [2], and this is predicted to increase up to 70% by 2050 [3]. AD is characterized by prominent neuroinflammation and reduced brain mass (Figure 1a) [4], which result in progressive decline in cognitive function, accompanied by neuropsychiatric symptoms, such as depression, anxiety, and even hallucinations [5]. While other major diseases (e.g., heart disease [6], cancer [7], and stroke [8]) reduce their mortality rate significantly, the deaths caused by AD continuously increase as the conventional AD therapies are merely relying on improving memory or alleviating psychotic symptoms for mild to moderate AD [3]. In addition, there are no available treatment options working for severe AD [9]. Therefore, it is an urgent issue to discover novel modalities preventing and curing AD [9].

To develop effective pharmacotherapeutic options for definitive cure of AD, enormous studies have explored the pathogenic mechanisms found in AD progression (Table 1). Primary pathological hallmarks of AD in the molecular level involve the accumulation of Aβ plaques [10,11] and neurofibrillary tangles (NFTs) [12,13], composed of dystrophic neurites, and hyperphosphorylated tau. These aggregates are gradually building up the intra and extracellular spaces of neurons, which block neurogenesis, as well as nutrient and oxygen supplies to neuronal cells, leading to neurodegenerative process [14]. In terms of the secondary characteristic, prominent activation of innate immune cells, such as astrocytes and microglia, is frequently found in pathogenic AD brain, which further induce excessive neurotoxic oxidative stress and proinflammatory mediators [15,16]. Importantly, the degree of neuroinflammation by innate immune cells statistically correlates with disease progression and severity in AD brains [17]. Given the significance, the targeting AD hallmarks has been deemed as an indispensable pipeline for developing therapeutic cures of AD.

In this review, we explore details on molecular and cellular mechanisms governing AD progression and their contributions to the development of current and new promising treatment modalities targeting mild to severe AD.

## 2. Central Hypothesis for AD Pathogenesis

### 2.1. Aβ Hypothesis

The formation of amyloid plaques from Aβ peptides is considered as a primary characteristic found in AD patients [10,81]. Under the physiological condition, the amyloid-beta precursor protein (APP) can be processed either by α-secretase followed by γ-secretase producing P3 fragments or by β-secretase and γ-secretase resulting in Aβ peptides [82]. Interestingly, the process by the combination of β-secretase and γ-secretase is dominant in the pathological conditions [83]. Depending on the cleaved region of APP by γ-secretase, the resultant Aβ can have majorly three isoforms as Aβ38, Aβ40, and Aβ42 [84]. Aβ42 is more hydrophobic form and prone to cause aggregates that are further forming oligomers, amyloid fibrils, and sequentially large proteinaceous deposits known as amyloid plaques (Figure 1b) [85]. Even if Aβ40 is the most abundant form, accounting for 80–90% of total Aβ, Aβ42 is the critical component to determine the severity of AD as the amyloid plaques derived from Aβ42 are known to cause neurotoxicity in AD progression [10,86].

Aβ hypothesis or amyloid cascade hypothesis, which was first introduced by Hardy and Allsop in 1991, emphasized on the significance of the Aβ deposition in the AD pathology [11]. Wang and co-workers examined AD patient brains and showed that the progressive shift of Aβ40 and Aβ42 from soluble to insoluble forms contributed to the AD progression [93]. The alteration in the APP metabolic pathway has been considered as one of the major underlying mechanisms of the Aβ deposition in AD pathogenesis [86]. For instance, the deposition of insoluble Aβ, particularly due to the mutations in the APP gene (e.g., KM670/671NL (Swedish), V717I (London), V717F (Indiana), and etc.), increased tendency of forming Aβ amyloid fibrils and plaques that further caused phosphorylation of tau, neuronal death, cell loss and dementia consequently [94]. In addition, mutations in the presenilin (PS) gene (e.g., PS1-M146L, PS1-L166P, PS1-I213T, PS2-N141I, and etc.) increasing Aβ42/Aβ40 ratios have been identified as causative factors of the AD plaques leading to the aggressive forms of AD. Other studies employing various APP/PS transgenic animal models recapitulating features found in the familial AD (FAD) supported the Aβ hypothesis by elucidation of the underlying mechanism of Aβ binding to neurons that caused the neuronal loss and cognitive decline [95,96]. Klementieva et al. discovered the significant accumulation of Aβ42 aggregates in synaptic compartments, which developed into Aβ plaques and induced the synaptic impairment in APP/PS1 mice brains, demonstrating that the deposition of Aβ on the synapsis initiated the AD pathology [97]. In addition, Aβ42, as well as Aβ 23–35, have been known to disrupt the blood-brain barrier (BBB) and further increase AD risk by binding to Receptor for Advanced Glycosylation End products (RAGE), down-regulating tight junction proteins, and increasing reactive oxygen species (ROS)-mediated damage in endothelial cells [98,99]. Moreover, recent advances in the optical microscopy technique have allowed us to detect non-fibrillar Aβ structures of the order of 100 nm and reveal their contributions to AD progression, suggesting the soluble to oligomeric Aβs could be more relevant targets to prevent neuronal loss and synaptic dysfunction [100,101]. Other recent studies have shown that the amyloid pathway can be modulated by tau [102], as well as innate immunity [103]. Pickett et al. found the cooperative works of Aβ and tau that decreased the expression of essential genes for synaptic functions in AAP/PS1 mice brains [102]. Hur et al. discovered the roles of interferon-induced transmembrane protein 3 (IFITM3) expressed in innate immune cells including astrocytes by using 5XFAD mice, which increased γ-secretase activity and promoted AD progression [103].

Despite the supporting evidence, several studies showed contradictory results, indicating that the Aβ hypothesis would not be the central cause of AD, particularly for the sporadic AD (SAD) cases [104]. For instance, Apolipoprotein E (APOE)-ɛ4, one of the major risk factors for SAD, is known to regulate the metabolism of Aβ contributing to Aβ depositions in the brain; yet the correlation of APOE-ɛ4 and AD progression is still controversial [105]. In addition, the Aβ hypothesis did not consider other risk factors for SAD, such as age and gender, to AD pathology [106,107]. Therefore, further AD studies should take into account such genetic and environmental backgrounds increasing the AD risks in order to understand AD pathology and discover novel therapeutic strategies properly.

### 2.2. Tau Hypothesis

Tau is a microtubule-binding protein, which stabilizes and modulates the microtubule assembly in the normal physiological condition. Upon the aggregation, tau is known to serve neurodegenerative functions in the pathological conditions, including AD, Pick disease (PiD), progressive supranuclear palsy (PSP), corticobasal degeneration (CBD), and frontotemporal dementia with parkinsonism-17 (FTDP-17) [108]. One of primary factors leading to the aggregation is the tau phosphorylation process on multiple sites, also referred as hyperphosphorylation [109] (Figure 1c). Among the six tau isoforms, two isoforms of tau possessing 4-repeat (4R tau) microtubule-binding domain and 3-repeat (3R tau) were found in a 50:50 ratio in normal brains, while the altered ratios by either increasing 4R tau or decreasing 3R tau were observed in the pathological conditions [12]. A number of studies suggested that the imbalanced ratio would result in the hyper-phosphorylation of tau, which further induced the accumulation of the insoluble filamentous form of tau, referred as paired helical filament (PHF) and NFT [12,110]. Tau having other tau modifications, such as proteolytic cleavage generating a fragment S258-I360 and O-GlcNac glycosylation in Serine/Threonine-Proline motif, was also prone to form tau aggregations [111,112]. The consequence of NFT formation involves the axonal degeneration, mitochondrial dysfunction, and synaptic dysfunction that further increase the neurodegeneration.

The tau hypothesis was first proposed by Kosik et al. in 1986, describing that the insoluble tau aggregates mediated by tau phosphorylation would be the central cause of AD [13]. In vitro studies revealed that abnormal phosphorylated tau proteins could increase the risk of AD significantly by disrupting microtubule stability and enhancing tau aggregates in the brain [88,113]. Although tau aggregations were predominantly expressed in neuronal exons, a recent study showed that tau aggregates in dendritic spines resulted in the synaptic impairment, as well [114]. In vivo studies also demonstrated that the capability of tau binding to synaptic vesicles localized in presynaptic terminals, resulting in abnormal neurotransmission, induced synaptic toxicity in rat neurons [115]. Several clinical studies have described the strong correlation between the tau pathology and the neurodegeneration leading to the loss of cognitive functions in AD conditions [116]. Bennett et al. reported the proportional relationship between the level of tau-positive tangles in neocortical regions and the significant cognitive impairment in AD patients [117]. Other clinical studies showed that NFTs were increased with age and affecting the entorhinal cortex, followed by the hippocampus, and destinating to the neocortex at the end [118]. In addition, the development of tau tangles was observed faster than that of Aβ aggregates [104]. Recently, Malpetti et al. revealed the strong correlation between tau pathology and neuroinflammation by using positron emission tomography (PET) imaging, indicating that the patterns of tau burden in posterior cortical regions and microglial activation in the anterior temporal lobes could predict cognitive declines in AD patients [119]. In addition, Simoes et al. screened proteomics of CSF modulated by endosomal trafficking and newly found two molecules, Amyloid-like protein 1 (APLP1) and close homolog of L1 (CHL1), which had strong correlation to the levels of tau and phosphorylated tau in CSF samples from AD patients [120]. Given the fact that the NFT development would be predictable, NFT pathology would offer an effective diagnostic method for AD at various stages, which may compensate the limitation found in Aβ hypothesis.

### 2.3. Oxidative Stress

Oxidative stress is an imbalanced situation between the levels ROS and antioxidants resulting in the accumulation of radicals in the cells increasing the risk of neuronal damages (Figure 1d) [121]. The ROS includes the non-radical oxidant as hydrogen peroxide radicals (H_2_O_2_) and the radical molecules as superoxide radical (O_2_^−^), hydroxyl radicals (·OH), and peroxynitrite (ONOO^−^) [121]. ROS production is majorly initiated by cellular metabolisms in organelles, including mitochondria, peroxisome, lysosome, endoplasmic reticulum, and plasma membrane [94]. Among the oxidative species, the superoxide and hydrogen peroxide are known to be the major sources to induce the oxidative damages in various brain disease models [122]. The accumulation of ROS can further initiate the production of reactive nitrogen species (RNS), as well as inflammation, in the central nervous system (CNS) [123]. In the normal conditions, the metabolic pathways of misfolded proteins or extracellular pathogens trigger the ROS/RNS production followed by the antioxidation maintaining homeostasis in brains serving neuroprotective roles [122,123]. These antioxidants involve the ROS scavengers (e.g., ascorbic acid (AA), proline, etc.), metal chelators (vitamins, N-acetylcysteine, alpha-lipoic acid, melatonin, etc.), antioxidant enzymes (e.g., superoxide dismutase (SOD), glutathione peroxidase (GPX), cytochrome oxidase, catalase (CAT), glutathione (GSH), etc.), and pro-oxidant enzyme inhibitors (e.g., nicotinamide adenine dinucleotide phosphate (NADP)-oxidase, cyclooxygenase (COX), lipoxygenase, etc.) [124]. Therefore, the disruption of the balance between metabolic pathway and protective system can increase the oxidative stress and the risk of CNS diseases.

The alterations in the metabolic mechanisms also significantly impact on the AD pathogenesis [124,125]. A number of studies showed that the alteration of Aβ clearance led to increasing Aβ burden, as well as oxidation of proteins, lipids, and nucleic acids, resulting in the increase of oxidative stress and neurotoxicity in AD hippocampus and cortex [126]. Recently, Chun et al. showed that oxidative stress driven by Aβ burden increased reactive astrocytes that further promoted tauopathy and exacerbated AD progressions in mice and human patient samples [127]. Mastroeni et al. also provided the evidence that oligomeric Aβ induced mitochondrial dysfunction modulating mitochondrial oxidative phosphorylation genes in the mild cognitive impairment (MCI) group [128]. Other studies revealed that tau tangles promoted deoxyribonucleic acid (DNA) and ribonucleic acid (RNA) oxidation increasing the AD risk as functional tau offered an essential safeguarding function preventing DNA and RNA oxidation [123,125]. In addition, the imbalanced antioxidant mechanisms in response to the metabolic stress can increase oxidative stress and lead to neuronal dysfunction and death sequentially [123]. Baldeiras et al. revealed that AD patients in the MCI group had the tendency to have the high level of lipid oxidation products along with the low level of antioxidant defenses (vitamins, GSH, uric acid, thiol groups, GPX, SOD, CAT), indicating the correlation between oxidative stress and dementia progression [129]. Recently, Youssef et al. elucidated the induction of antioxidant mechanisms, mediated by Nuclear factor erythroid 2-related factor (Nrf2) and heme-oxygenase (HO), in the superior temporal gyrus under the early stages of AD from young and APOE e4-matched AD patients [130]. Moreover, recent reports validated the significant roles of metal ions (e.g., Zinc, Copper, etc.) modulating the activity of antioxidant enzymes, pointing out that the disruption of metal homeostasis contributed to the oxidative stress increasing neurotoxic effects in brain of AD [131]. Therefore, oxidative stress has been considered as a risk factor for AD.

### 2.4. Neuroinflammation

The neuroinflammation hypothesis emphasizes that the detrimental immune response plays a central role in the dysregulation of neuronal functions and the development of CNS diseases [15] (Figure 1e). Microglia and astrocytes are two major components of the resident immune cells involved the inflammatory responses in the brain disorders [132]. Microglia, resident myeloid cells of a CNS, continually survey their microenvironments in normal and diseased brains, provide immune surveillance, and are activated in response to infection, non-infectious diseases, and injury [15]. They play neuroprotective roles by phagocytosis and clearance of damaged synaptic parts or dead neurons [133]. Astrocytes are the most abundant glial cells, also serving neuroprotective roles by secretion of neurotransmitters supporting neuronal activity and removal of debris and toxins around neurons, as well as in cerebrospinal fluid (CSF) [16]. Both microglia and astrocytes are changing their phenotypes promoting the detrimental proinflammation under the CNS disease conditions, particularly in the severe conditions [134]. In this regard, the incompetent activation of immune cells in mild conditions or the exaggerated activation in severe environments would increase the risk of multiple CNS disorders, including AD, Parkinson’s Disease (PD), Amyotrophic Lateral Sclerosis (ALS), and dementia [135].

Prominent activation of innate immune cells, followed by the marked neurotoxic neuroinflammation, have been observed during AD progression [136]. While such responses can be protective against Aβ plaques and/or tau aggregates in the early stage [137], the neuroinflammation may also be detrimental to patients by neurotoxic inflammatory mediators leading to brain dysfunctions within the neocortex and hippocampus in the late stage of AD [138]. Weldon et al. showed that proliferation followed by activation of microglia and astrocytes were observed around Aβ fibrils and plaques, which precipitated the production of proinflammatory factors, such as tumor necrosis factor-α (TNF-α), interleukin (IL)-1β, and IL-18, in the AD brain models [134]. In accordance with in vitro and in vivo AD models, clinical studies provided the supportive evidence of high population of reactive astrocytes and microglia exerting neurotoxic activities in AD microenvironments [139]. AD patients exhibited the high levels of cytokine, such as IL-12, IL-16, IL-18, and transforming growth factor beta (TGF-β), in CNS accompanied by the axonal degeneration and neuronal death [140]. Indeed, the increase in TNF-α, IL-1β, IL-12, IL-18, and TGF-β were frequently observed in the late stage of AD [141]. Compared to the Aβ and tau hypothesis, the neuroinflammation could describe the correlation between the reactivity of innate immune cells and the severity of clinical symptoms well representing the effective mechanisms further contributing to the development of new diagnostics and/or therapeutic strategies [142].

The innate immunity further contributes to activate other immune systems in CNS, such as adaptive immunity. Under neuroinflammatory conditions, adaptive immune cells residing in the peripheral blood can be infiltrated into the CNS region and involved in the AD progression in response to soluble factors released by innate immune cells [143]. Both T helper cells and cytotoxic T cells were detected in post-mortem brain slices of AD patients, frequently found near plaques or tangles [144]. Several studies suggested that the crosstalk of T cells with other glial cells in CNS would be an essential prerequisite to obtain fully activated phenotypes under AD microenvironments as the infiltrated T cells were initially not activated or on the progress of maturation [145]. Furthermore, reactive glial cells in AD can further recruit and activate B-lineage cells [146]. The increased population of activated B cells producing antibodies against Aβ and tau aggregates were found in AD patients, which directly disassembled Aβ and tau proteins and further assisted the microglial phagocytosis [147]. Even if primary roles of T cells and B cells are to maintain homeostasis in mild AD conditions, the crosstalk between glial cells and these peripheral immune cells can increase the production of proinflammatory mediators (e.g., IL-1β, IL-6, IL-12, IL-18, and TNFα), leading to neurodegeneration in severe ADs [148]. Therefore, targeting innate immunity along with peripheral immunity can be effective strategies to cure mild to severe ADs.

## 3. Promising Strategies for Targeting Each Stage of AD Development

### 3.1. Conventional Strategies Targeting Aβ

#### 3.1.1. Inhibition of Aβ Cascade

The β-secretase enzyme (BACE)-1 inhibition has been a pharmacological strategy for AD as the cleavage of APP by BACE-1 is the first step in the production of soluble Aβ [149,150,151,152,153,154]. Verubecestat and Lanabecestat are well-studied BACE-1 inhibitors in animals and have proven their effectiveness in mild to moderate AD patients, such as reduction in soluble Aβ in CSF, significantly [150,151]. However, the reduction in Aβ levels in CSF did not improve either cognition or brain functionality; yet, even worsening of cognition has been reported [144]. Other BACE-1 inhibitors (e.g., Elenbecestat [152], Atabecestat [153], CNP520 [154]) are under the clinical evaluation in terms of the reduction of Aβ accompanied by the deceleration of cognitive decline. Blocking γ-secretase pathways (e.g., Tarenflurbil [23], Semagacestat [22], Avagacestat [155]) is another approach in decreasing APP proteolysis followed by Aβ production, but they were not able to improve cognitive functions of AD brains neither [23]. Other approaches, such as increasing α-secretase activity and modulating lipid metabolism reducing APP-mediated Aβ cascade, are under the stage of development [149].

#### 3.1.2. Passive Immunotherapy Targeting Aβ

The amyloid cascade hypothesis presents that the most neurotoxic species is the oligomeric Aβ as it elicits the disruption of neuronal membranes, the dysfunction in synapsis, and the signal transduction initiating process of AD [11,93,94,95,96]. Recent evidence showed that the formation of oligomeric Aβ was a reversible process that reduced the neurotoxicity in various brain models [156]. Therefore, the anti-Aβ antibodies destabilizing or clearing Aβ oligomer have been a strategy for treating the mild to moderate AD [25,26,157]. Particularly, N-terminus of Aβ-targeting antibodies showed the excellent effectiveness to clear the toxic aggregated species of Aβ [157]. Among the humanized monoclonal immunoglobulin G (IgGs) targeting N-terminus, Bapineuzumab and Gantenerumab have proven their outstanding binding affinity to oligomeric Aβ, but they failed to improve cognitive functions in clinical trials [25,26]. To further improve the therapeutic efficacy, Biogen screened libraries of memory B cells from healthy elderly and released Aducanumab, a N-terminus targeting antibody targeting oligomer and fibril types of Aβ [158]. Promising animal studies showed that Aducanumab can penetrate the blood-brain barrier (BBB), while other antibodies achieved the penetrate rate of 0.1% [158]. Furthermore, clinical studies validated the therapeutic effectiveness of Aducanumab slowing of progression at 1 year; and now it is under Phase III [126]. Polyclonal antibodies or immunoglobulins are also capable of decreasing Aβ burden in the brains, but their major mechanism to treat AD is the active immunization that recognizes Aβ species and boosts the immunological response against Aβ [159]. The details will be described in Section 3.3.

#### 3.1.3. Inhibition of Aβ Aggregation

The metal protein attenuating compounds (MPACs) have emerged as potential anti-Aβ aggregates as they strongly chelate excessive copper and zinc ions and inhibit the interactions between redox-active ions and Aβ peptides that further prevent oligomerization [27,160,161,162]. Clioquinol (PBT1) is a hydroxyquinoline ionophore, exhibiting the excellent inhibition of Aβ deposition in AD mice models and the decrease of Aβ toxicity in neuronal cell culture models [160]. Clinical studies proved that the treatment of Clioquinol significantly reduced the Aβ42 level in plasma and benefited for the patients having mild cognitive impairments (Alzheimer’s Disease Assessment Scale-Cognitive Score (ADAS-cog Score) > 25) compared to the treatment of placebo drug [161]. However, there was a rising safety issue, such as neuropathy in the patients with the chronic administration of Clioquinol [162]. A second-generation of clioquinol (PBT2) with the improved brain penetration and pharmacokinetics was developed and confirmed the safety maintaining synaptic health in APP transgenic mice. Currently, PBT2 is under the clinical evaluation [27].

#### 3.1.4. Challenges

Preventing the Aβ accumulation was the major approach to treat the mild to moderate AD for the past two decades (Table 1). Despite the efforts for the diminution of Aβ deposition in the brains using above mentioned strategies, no promising clinical outcomes has been accomplished [18,25,26,162]. Due to the clinical failures, a number of studies casted doubt on the current Aβ hypothesis suggesting that Aβ accumulation would be merely an epiphenomenon not a major cause of AD progression [104,163,164]. For instance, the contradictory results have been reported that Aβ aggregates can be formed in even healthy brains, indicating the implausible correlation between the accumulation of Aβ and the severity of AD [165]. In addition, the reduction of Aβ in CSF or Aβ aggregation in brains did not improve cognitive functions but frequently increase adverse side effects in the human body [150,151]. Moreover, Aβ-targeting strategies are limited to the early to moderate AD patients [18,23,43,89,166] and are not available for the late-onset AD patients, who have the high risk of cognitive impairment, dementia, or even lose control of essential bodily functions [104,163,164]. Given the risk-benefit ratio, most Aβ-targeting strategies have been under reconsideration or even discontinued in their use [165].

### 3.2. Inhibition of Tauopathy

#### 3.2.1. Inhibition of Hyperphosphorylation in Tau

As we described in the Section 2.2, the hyperphosphorylation of tau (more than 7–8 phosphates per a tau) induces the detachment of tau from microtubules leading to the formation of tau aggregates and insoluble filaments in the diseased state [167]. Hyperphosphorylation is modulated by several protein kinases, including glycogen synthase kinase-3β (GSK-3β), cell cycle-dependent kinase 5 (CDK5), mitogen-activated protein kinases (MAPK), casein kinases, Ca2+/calmodulin-dependent protein kinase II (CAMK II), microtubule affinity regulating kinase, and protein kinase A (PKA) [167,168]. Among the kinases, GSK-3β and CDK5 were observed near neurofibrillary tangles promoting the microtubule aggregates in AD patients [168,169]. Other in vitro and in vivo results showed that the inhibition of GSK-3β and CDK5 activity were capable of reducing tauopathy and degeneration by lowering tau phosphorylation [170]. Thus, GSK-3β and CDK5 can be novel targets for a therapeutic intervention of AD, as well as other CNS disorders [168,170]. For example, alkali metal lithium and amino-thiazole, GSK3 inhibitors, proved their therapeutic efficacy to reduce tau phosphorylation in transgenic mouse and human AD models [170]. In addition, inhibitors targeting MAPK or CAMKII have received a great attention as promising therapeutic approaches of AD by reducing the tau-mediated aggregates in the brain [171]. Other examples of inhibitors decreasing the hyperphosphorylation are 4,5,6,7-tetrabromobenzotriazole (TBB) (casein kinase II inhibitor), cyclosporin A (calcineurin inhibitor), and Saracatinib (Fyn kinase inhibitor) [172].

The usage of tau phosphatase can be another strategy to prevent the formation of tau hyperphosphorylation [173]. Protein phosphatase 2A (PP2A) is a large family of phosphatases targeting Ser/Thr protein kinases in the brain, which is capable of dephosphorylation and inactivation of various tau kinase proteins [173,174]. The reduction of PP2A activity was found in the brain of AD patients, promoting tau hyperphosphorylation [175]. Besides PP2A, other protein phosphatases, such as PP1, PP2B, and PP5, also significantly downregulated the tau phosphorylation [176]. However, Liu et al. showed that PP2A, PP1, PP5, and PP2B accounted for 71%, 11%, 10%, and 7% of the total dephosphorylation, respectively [177]. Thus, the activation of PP2A can be a pharmacological approach to inhibit the hyperphosphorylation. The small molecules regulating PP2A have been developed and proved their ability to deregulate tau hyperphosphorylation and reduce the cognitive impairment consequently [177]. For example, the copper ionophore glyoxalbis-(N4-methylthiosemicarbazonato]-Cu(II) (Cu^II^(gtsm)] improved PP2A activity and provided benefits towards the tau pathology in animal model [178]. Other metal chelators targeting zinc or iron also promoted the PP2A activity that further reduced tau phosphorylation level. However, a number of clinical studies reported issues with the long-term usage of some phosphatases (e.g., perphenazine (PPZ) and okadaic acid (OKA)) causing the neurotoxicity [179]; therefore, the application of small molecules in the treatment of AD requires further research.

#### 3.2.2. Inhibition of Tau Aggregation

The formation of tau aggregations, also referred as NFTs, are the consequence of misfolded tau accumulation followed by PHF formation [180]. Therefore, the inhibition of aggregation process or the destroy of aggregated tau could be effective therapeutic approaches preventing tauopathy in AD progression [181]. To impede the formation of tau aggregates, small molecules disturbing tau-tau paring have been developed achieving 80% disassembly of PHF in the brains [182], proving their therapeutic efficacy as AD treatments. Methylene blue (MB) is one of the first-generation inhibitors disrupting tau aggregations and serving neuroprotective roles via promotion of mitochondrial antioxidants, which is now in phase II clinical trials for treating mild AD [183]. To solve undesirable side effects (diarrhea, urgency, and painful urination) reported in MB treatment, a new MB derivative (leuco-methylthioninium bis (hydro-methanesulfonate) (LMTM)) has been designed to improve the bioavailability and tolerability [184]. Recently, LMTM has been proven the ability to decrease tau aggregation and improve cognitive impairment in the phase III clinical trials [184]. Other small molecules preventing tau aggregation involve thiacarbocyanines, anthraquinones, and phenylthiazolyl-hydrazides, which have been demonstrated their promising therapeutic efficacy in vitro and in vivo AD models [184,185]; yet, further investigation is required.

#### 3.2.3. Inhibition of Tau Activity

The inhibition of tau expression level and activity can alleviate AD symptoms [167]. The currently proposed treatments inhibiting the tau activity include tau expression inhibitors (antisense oligonucleotides (ASOs)) [28], phosphatase modifiers (e.g., PPZ, OKA, Memantine, etc.) [29,30,31,32,33], tau kinase inhibitors (e.g., Tideglusib, lithium, amino-thiazole, oxindolequinazoline, sirenade, R-roscovitine, aloisine, etc.) [34,35,36,37,38,39,40], tau acetylation inhibitors (Salsalate) [41], tau deglycosylation inhibitors (e.g., phosphodiesterase type 4 inhibitor) [42], tau aggregation inhibitor (e.g., Methylene Blue, LMTX, etc.) [43,44,45,184,186], microtubule stabilizers (e.g., Epithilone D, NAP, TPI 287) [47,48,49], and small molecules promoting immune response toward tau [178].

#### 3.2.4. Challenges

Given the fact that the tau burden explained the clinical impairments better than Aβ burden did, tau protein is considered as a promising target for AD [167]. However, the above-mentioned medicines are not capable of curing AD, but only relieving mild symptoms, presumably due to lack of knowledge on tauopathy [104]. For instance, the contribution of tau to Aβ pathology, or vice versa, in AD progression is not fully understood [164,167]. Therefore, the further research on the underlying mechanisms of tau dysfunction and their impact on AD progression is essential to discover novel AD cures.

### 3.3. Neuroinflammatory Modulation

The conventional treatments were merely targeting early ADs reducing symptoms. To prevent AD progression particularly targeting the late stages of ADs, the reduction of the glial activity by removal of excessive proinflammation has proven notable outcomes recently [187]. The glial activation is attributed to the binding of misfolded proteins or pathogens to Toll-Like Receptor (TLR), Triggering Receptor Expressed on Myeloid cells 2 (TREM2), RAGE, Colony-Stimulating Factor-1 Receptor (CSF1R), or P2Y1 purinoreceptor (P2Y1R) [188]. Upon the activation of microglia and astrocytes either by misfolded proteins or proinflammatory cytokines, the signal transduction via Janus Kinase (JAK)-Signal Transducer and Activator of Transcription (STAT), MAPK, Nuclear factor-κB (NF-κB), or Nod-Like Receptor (NLR) family Pyrin domain containing 3 (NLRP3) is initiated and further increased the inflammation and neurodegeneration in CNS [189]. Therefore, inhibition of inflammatory response targeting one of these cascades can be a novel strategy to reduce detrimental neuroinflammation and cognitive deficits induced by AD.

#### 3.3.1. Inhibition of JAK2/STAT3 Pathway

The binding of Aβ to gp130 membrane receptors and triggering the phosphorylation of STAT3 and JAK/STAT3-associated signaling pathways are commonly found in the reactive astrocytes and microglia under the AD conditions [190]. The phosphorylated STAT3 are then turning on the expression of targeted genes, resulting in the up regulation of proinflammatory mediators, such as IL-6, IL-12, TNF-α, and interferon gamma (IFN-γ) [191]. The constitutive activation of JAK2/STAT3 pathway by mutation of JAK2^T875N^ induced the increase of reactive astrocytes in CNS and altered synaptic transmission [192]. Therefore, the downregulation of JAK2/STAT3 can alleviate AD pathology, particularly targeting reactive astrocytes. For instance, inhibition of the cascades by the treatment of Suppressor of Cytokine Signaling 3 (SOCS3) inhibitors [192] or the conditional deletion of STAT3 [193] lowered the expression of reactive marker of glial fibrillary acidic protein (GFAP) and restored the transcription profile of astrocytes under the inactive status [192,193]. JAK2/STAT3 inhibitors also rescued the synaptic transmission and plasticity that improved memory and learning functions in AD mice. Oral administration of Stattic, a STAT3 inhibitor, has proven the therapeutic efficacy to recover learning and memory in 5XFAD mice model [193,194].

#### 3.3.2. Inhibition of NF-κB/NLRP3 Pathway

NF-κB is another transcription factor triggering NLRP3 inflammasome cascades, which produces proinflammatory molecules as IL-6, IL-1β, and IL-18 [194]. The abnormally increased NLRP3 activity via NF-κB, which exacerbated neuroinflammation, was frequently found in the AD pathology [195]. In vitro studies showed that the accumulation of Aβ can activate the NF-κB/NLRP3 pathway in reactive astrocytes and lead to the production of excessive IL-6, which further triggered γ-Aminobutyric Acid-producing (GABAergic) interneuron degeneration and cognitive impairment [196]. Valerio et al. showed that injection of Aβ1-42 oligomers to the cortex of rat increased the colocalization of NF-κB and GFAP, validating the involvement of Aβ-mediated NF-κB activation in the increase of astrocyte reactivity [197]. Other studies also revealed that reactive astrocytes released IL-1β via the NF-κB-mediated pathway led to the tau hyperphosphorylation and the tangle formation, indicating that NF-κB/NLRP3 increased AD risk [198]. Alternatively, in vivo studies with APP/PS1/NLRP3^--/--^ mice pointed out the opposite effects of NLRP3 deficiency that increased microglial phagocytosis and skewed microglia into anti-inflammatory phenotype serving neuroprotective roles [195]. In this regard, inhibition of NF-κB/NLRP3 cascades exerted either by removal of NF-κB-mediated proinflammation or addition of NLRP3 agonists and can be employed for AD treatment [199].

#### 3.3.3. Inhibition of p38 MAPK Pathway

p38 MAPK is emerging as a pivotal regulator for promoting proinflammatory pathways. Extracellular signals, such as Aβ, can initiate p38 MAPK cascades in microglia and astrocytes, leading to the release of proinflammatory molecules, particularly IL-1β, exacerbating AD pathogenesis [171]. The activation of p38 requires the binding of adenosine triphosphate (ATP) followed by the phosphorylation of threonine at R180 and tyrosine at R182 [200]. From the perspective of molecular mechanism, p38 MAPK inhibitors preventing the binding of p38 to ATP have been developed: one for targeting ATP-competitive mechanism, such as imidazole inhibitors (SB203580, SB202190, SP600125), and another for changing conformational structures of ATP-binding sites (BIRB796) [201]. These p38 MAPK inhibitors can prevent the formation of BACE-1 protein, induce the expression of Aβ-degradable enzyme (neprilysin), and decrease pro-inflammatory mediators [202]. Another type of selective p38 MAPK inhibitor, NJK14047, down-regulated the expression of proinflammatory factors, such as COX-2, TNF-α, IL-1β, and nitric oxide (NO) synthase, subsequently leading to a reduction of NO and prostaglandin E2 [203]. In this regard, the negative regulation of MAPK can reduce Aβ accumulation, alleviate neuroinflammation, and improve cognitive impairments in AD mice [204].

#### 3.3.4. Calcium/Calcineurin/NFAT Pathway

Dysregulation of glial Ca^2+^ signaling was found in a number of CNS diseases, including AD, brain edema, stroke, and epilepsy [205]. In AD, the presence of Aβ and proinflammatory factors originated from microglia and astrocytes increased the excessive calcium pools and triggered the Calcium/Calcineurin/Nuclear Factor of Activated T-cells (NFAT) signaling pathway, as well as the production of proinflammatory cytokines that exacerbated AD progression [206]. The inhibition of calcineurin/NFAT pathways were particularly effective for the reduction in astrocyte reactivity, while baring for other innate immune cells. For instance, blocking the calcineurin/NFAT axis in astrocytes by using VIVIT peptide reduced the reactivity and amyloid levels, as well as improved the cognitive deficits, in APP/PS1 mice [207]. Chronic treatment with calcineurin inhibitors, such as Tacrolimus, showed a neuroprotective effect on the AD compared to general population [55,207,208]. However, inhibiting this pathway in microglia did not offer a benefit to improve memory performances although the Aβ load and microgliosis were significantly reduced [55]. Overall, the maintenance of calcium homeostasis in CNS by modulating calcineurin/NFAT pathways can offer benefits to ameliorate AD progression.

#### 3.3.5. TREM2 Pathway

TREM2 is a membrane receptor primarily expressed in microglia sensing Aβ or other toxic aggregates in AD [209]. Recently, the deficiency of TREM2 activity has been identified as a high-risk factor as it has a strong correlation with the severity of AD. Several studies validated that the deficiency of TREM2 increased proinflammatory responses [210], while recovery or overexpression of TREM2 promoted anti-inflammatory responses [211]. In addition, TREM2 receptor is a checkpoint for the activation of type II Disease-Associated Microglia (DAM2), which is responsible for the clearance of Aβ plaques in hippocampus, cerebral cortex, amygdala, and hypothalamus [212]. Therefore, the deficiency or haploinsufficiency of TREM2 impaired the activation of neuroprotective microglia and increased the Aβ burden, as well as the cognitive impairment [211,213]. Multiple genetic mutations, such as APOE, R47H, R62H, D87N, and T96K, in TREM2 are related to malfunctions in phagocytosis and promotion of proinflammatory activation [214]. For instance, R47H variant of the gene encoding TREM2, which is most abundant mutation in AD patients comprising 75% [215], induced the loss of TREM2 function, contributing to the suppression in synaptic transmission [216] and the increase of proinflammatory immune response exacerbating AD risks [217]. Given the important roles of TREM2 in AD, therapeutic strategies to recover or promote TREM2 functions have been intensively investigated [218]. Treatment of additional soluble TREM2 (sTREM2) or viral vectors increased the microglial phagocytosis and rescued spatial memory [219]. Overexpression of TREM2 in microglia by using Bacterial Artificial Chromosome (BAC)-mediated transgenesis can reduce the Aβ load and improve memory performance in 5xFAD mice model [220]. Antibodies that rescued the loss of TREM2 signal were developed and proved their therapeutic efficacy that can decrease the pro-inflammatory cytokines and abrogate survival defects [57].

#### 3.3.6. TLR Pathway

TLRs are a family of pattern-recognition receptors expressed in a wide spectrum of CNS cells, particularly microglia [221]. The involvement of TLRs, especially TLR2 and TLR4 in AD pathogenesis, have been investigated in many studies; however, their specific roles in the initiation and progression of AD remain controversial [222]. In vitro studies with mouse-derived microglia showed that the activation of TLR2 was responsible for Aβ [223]. However, other studies on APP/PS1 mice reported the contradictory findings that inhibition of TLR2 reduced Aβ load and improved impairments in spatial learning functions [224]. Like TLR2, there are still controversial debates over the role of TLR4 in AD pathogenesis [222]. Studies on APP/PS1 mice with a destructive mutation of TLR4 reported that the loss of function in TLR4 was associated with the increase in the Aβ deposition and the decrease in the microglial amyloid beta hypothesis activation, while the activation of TLRs significantly boosted the Aβ phagocytosis [225]. On the other hand, recent studies showed that the activation of TLR4 by Aβ42 increased TNF-α level, leading to the long-term potentiation deficit and neuronal death [226]. These inconsistent findings suggest that more studies on the roles of TLRs in AD are in need to develop AD treatments.

#### 3.3.7. RAGE/CSF1R/P2Y1R Pathway

Overexpression of RAGE in microglia leads to the increased production of neuroinflammatory cytokines and Aβ accumulation; therefore, the treatment of RAGE inhibitors can prevent the exasperated reactivity of microglia towards Aβ [227]. CSF1R is also a promising target to regulate the inflammatory response of microglia [59]. Prolonged inhibition of CSF1R by using GW2580 tyrosine kinase inhibitor can shift microglia from inflammatory phenotype to anti-inflammatory phenotype [228]. Furthermore, chronic intracerebroventricular administration of P2Y1R antagonists, which notably prevented the hyperactivity in astrocytes, protected the brain from impaired spatial learning and memory deficits [60].

#### 3.3.8. Anti-Inflammatory Therapy Targeting Adaptive Immune System

Beneficial roles of T cells (Tregs) and type II helper T cells (Th2) in AD have been reported in many studies [61,229,230]; therefore, the increase of T reg or Th2 population has been adopted as a new clinical approach for AD treatment. Transplantation of Treg cells, also referred as adaptive T cell therapy, reduced Aβ burden and recovered cognitive functions, while deletion of Treg population significantly increased Aβ load and exacerbated AD progression, with spatial learning deficits [61]. Treatment of Aβ-specific Th2 lymphocytes also improved the memory working tasks in APP/PS1 mice models [61]. One of the major advantages of T cell transfer therapy compared to others is that T cells are inherently passing through BBB, not requiring additional delivery system [231]. B lymphocytes, another type of adaptive immune cells, increased anti-Aβ IgG, which neutralized and impaired Aβ fibrinogenesis [232]. Loss of B cells induced the impairment of microglia phagocytosis, while the addition of anti-Aβ IgG could compensate the reduction effects in Aβ pathology [62]. Type I helper T cells (Th1), on the other hand, is known to promote proinflammatory cytokines (IFN-γ, TNF-α, IL-2) [166,233] resulting in detrimental consequences. These contradictory roles in the adaptive immune system depending on subtypes of lymphocytes indicate that therapeutic approach should target and control over each type of lymphocyte specifically so that increase the success rate of immune therapy.

#### 3.3.9. Challenges

The anti-inflammatory treatments have received great attentions recently due to the notable correlation between the degree of neuroinflammation and the severity of AD [127,146,234] (Table 1). On the other hand, there are only few treatments in the clinical trials (e.g., ALZT-OP1, COR388, masitinib, etc.) [235,236,237]. In addition, Non-Steroidal Anti-Inflammatory Drugs (NSAIDs) did not validate convincing efficacy in clinical trials, although they showed promising therapeutic efficacy in in intro and in vivo models [15]. The clinical failures were attributed to the inaccurate targeting of the specific component in innate immunity as innate immune cells were heterogeneous in their phenotype and played dual roles depending on the stage of AD [238]. For instance, microglia and astrocytes played neuroprotective roles in the early onset of AD, but they then switched to the detrimental phenotypes exacerbating neuroinflammation in the late stages of AD [236]. In addition, it required significant challenges to determine the stage of AD where the glial cells were shifting from neuroprotective to neurotoxic phenotype [238]. Therefore, further studies on the characteristics of innate immune cells and their mutual interactions in each stage of AD are urgently required.

## 4. Neuroregeneration Restoring Cognitive Impairment

### 4.1. Supplement of Neurotrophic Factors

Neurotrophic factors are biomolecules playing key roles in the proliferation, differentiation, plasticity, and survival of neuronal cells [239]. Neurotrophic factors are composed of three main families, including neurotrophins, glial cell line-derived neurotrophic factors (GDNFs), and ciliary neurotrophic factors (CNTFs) [239]. Among the neurotrophic factors, the deficiency or dysregulation of neurotrophins is the hall marker prevailing in AD patients resulting in severe neuronal defects [237]. Neurotrophin family composes of Brain-Derived Neurotrophic Factor (BDNF), Nerve Growth Factor (NGF), Neurotrophin (NT)-3), and NT-4. The roles of neurotrophins are initiated by binding to Tropomyosin Receptor Kinases (Trks) or p75^NTR^, which transduces signaling pathways of Phosphatidylinositol 3-Kinase/protein kinase B (PI3K/AKT) and MAPK–Extracellular signal Regulated Kinase (ERK) promoting protein synthesis, cell proliferation, and cell survival [240]. Clinical studies revealed that the levels of neurotrophins in AD patients were increased in early stages, while their levels were significantly decreased in advanced stages [241]. In this regard, the supplementary of neurotrophins can be a therapeutic strategy for treating AD.

#### 4.1.1. Supplement of Neurotrophins

The restoration of neurotrophin deficiency can reverse neurodegenerative diseases [242]. The supplementation of neurotrophins, particularly BDNF, to the hippocampus region has proven the efficacy to ameliorate learning deficits in the rat model of AD induced by the treatment of Aβ1-42 [63]. However, recent studies showed that the untargeted delivery of NGF continuously stimulated neuronal cells causing constant neuropathic pains for patients several months after infusion [243]. To this end, targeted drug delivery system and painless NGF protein (hNGFp) have been suggested to solve this issue, which proved the restoration of cognitive deficits without causing neuropathic pains in animal models [65,244]. The supplementation of NGF can be further exerted by the delivery of NGF encoding vectors to CNS regions [245]. The NGF genes were inserted in viral vectors, such as Moloney leukemia virus vectors and Adeno-associated virus serotype 2 (AAV2), and tested to treat mild-to-moderate AD patients [65].

#### 4.1.2. Increase of Neurotrophic Effects by Peptide Mimetics

The indirect strategy to increase neurotrophic effects is the delivery of peptide mimetic, small molecule ligands that target neurotrophin receptors (either TrkA, TrkB, TrkC, or p75^NTR^) in the agonist or antagonist manner [246]. For instance, the peptide antagonist of NGF targeting p75^NTR^ prevented the neuronal death induced by Aβ (1–40) in NIH--3T3 and E17 cortical neurons and improved the cognitive deficits in APP mice [244]. 7,8-dihydroxyflavone (7,8-DHF) is another peptide mimicking BDNF blocked BACE-I receptor and alleviated the level of Aβ40 and Aβ42 in 5XFAD mice brains [66]. 7,8-DHF also transduced the TrkB-axis pathways that improved cognitive deficits and prevented neuronal loss in AD mice model [67,247] without causing toxicity in long-term treatment. Other examples involve deoxygedunin [67], LM22A-4 [68], and bicyclic BDNF loop mimetics [248] that have proven their BDNF neurotrophic effects in AD.

In summary, neurotrophin dysfunction is closely associated with the pathogenesis of AD. Enhancing the neurotrophin effects could be achieved by the direct supplementation of neurotrophins or by the indirect addition using gene therapy and neurotrophin mimetics. Although there are remaining challenges in these methods, current results suggest that neurotrophin therapy is a promising strategy for the treatment of AD.

### 4.2. Supplement of Neuronal Cells

Another strategy to promote the neuroregeneration is the supplement of functional neurons, which are capable of forming new axons, synapses, and neural networks in the damaged CNS regions [249,250]. In order to increase population of functional neurons, neurons can be supplemented either by the glia-neuron conversion or the stem cell transplantation [251,252]. Glial cells are essential in the survival and the maintenance of normal neuronal function, such as supplying nutrition to neuronal cells [253,254], promoting myelination, and maintaining synaptic connectivity and plasticity [255,256]. In addition to the benefits to repair and promote neuronal activity, these glial cells can be converted into different subclasses of neuronal cells accompanied with their original roles [70]. Therefore, several studies have developed the glia-neuron conversion techniques that may offer a novel strategy to cure AD [72,73,257,258]. Stem cell transplantation is another emerging approach that reverses the pathogenesis of AD [259]. The transplanted stem cells are differentiated into newly generated neuronal cells that replace the damaged neurons and recover dysfunction in APP/PS1 mice brains [260]. The newly generated neuronal cells can be further genetically modified to produce neuroprotective factors promoting self-repair system in endogenous cells [74]. In addition, the replaced stem cells are known to decrease the proinflammation contributing to the exacerbation of AD in the cortex and hippocampus [70,257]. Currently, those two approaches are under the clinical trials to demonstrate their safety, which may pave the way for the development of a novel approach to cure AD.

#### 4.2.1. Conversion of Glial Cells to Neuronal Cells

The promoted expression of neuronal transcription factors can convert each type of glial cells to the different subclass of neuronal cells, which may offer the replacements of impaired neurons [261]. For example, under the forced expression of NeuroD1 by using viral vectors, astrocytes were converted into glutamatergic neurons, while NG2 cells became GABAergic neurons [257]. The converted neurons were fully functional showing robust synaptic activities validated by the cortical recordings [257]. Moreover, by using different transcription factors, a particular type of glial cell can be programmed to convert into different types of neurons [70]. To take astrocytes as examples, astrocytes derived from postnatal cortex could be transformed into either glutamatergic or GABAergic neuronal cells by using the fate determinant Neurogenin 2 or Dlx2 [70]. The conversion efficacy also depends on the origin of glial cells. Astrocytes from grey matter were reprogramed and converted into functional neurons more effectively than that of white matter [71]. The addition of chemicals targeting transcriptional factors can also facilitate the glia-neuron conversion [262]. Zhang et al. introduced the method of glia-neuron conversion by addition of nine small molecules sequentially, including LDN193189, SB431542, 4-((E)-2-(5,6,7,8-Tetrahydro-5,5,8,8-tetramethyl-2-naphthalenyl)-1-propenyl)-benzoic acid (TTNPB), thiazovivin, CHIR99021, valproic acid (VPA), N-(N-(3,5-difluorophenacetyl)-L-alanyl)-S-phenylglycine t-butyl ester (DAPT), Smoothened agonist (SAG), and purmorphamine, that were capable of activating neural transcription factors, such as NeuroD1 and Neurogenin 2 [72]. Later, Yin et al. developed a more simplified recipe of four molecules, LDN193189, CHIR99021, DAPT, and SB431542, that modulated multiple signaling pathways, including Notch, which was simultaneously converting astrocytes to neurons by one-time administration [73]. Interestingly, the new cocktail was found to pass through the BBB and induced neurogenesis in the mouse hippocampus [73]. The successful conversion of glial cells into functional neurons may lead to the development of next-generation medicines for neuroregeneration.

#### 4.2.2. Transplanting Stem Cells into Brains

Mesenchymal stem cells (MSCs) are the most widely used stem cells for the replacement of impaired neuronal cells for AD patients [263]. MSCs could be derived from a variety of tissues and organs, such as adipose tissue, bone marrow, placenta, or umbilical cord; particularly, MSCs isolated from adipose tissue showed an excellent proliferation rate [264]. MSCs are more appealing compared to other types of stem cells for neuroregeneration as they are inherently capable of passing the BBB layer [265]. This unique characteristic enabled the administration of MSCs in the less invasive routes, leading to the simplification of treatment protocol [266]. In addition to MSCs, neural stem cells (NSCs) are the second major source for the stem cell transplantation in AD treatments [267]. NSCs can be isolated from the subventricular zone of lateral ventricles or the subgranular zone of the hippocampus [268]. They have the capability of differentiating into oligodendrocytes, neurons, and astrocytes [269]. Other types of stem cells, such as embryonic stem cells, hematopoietic stem cells, olfactory ensheathing cells, and induced pluripotent stem cells, have been also employed in the treatment of AD [74]. To further improve the efficacy of transplantation followed by AD cure, these stem cells were genetically modified to encode neurotrophic factor genes [76,77,78,79] or pretreated with neurotrophic factors [76] insulin-like growth factor-I (IGF-I) [77], Vascular Endothelial Growth Factor (VEGF) [78], or Glucagon-Like Peptide-1 (GLP-1) [79]. For example, BDNF electroporated into mouse NSCs increased the viability of engrafted NSCs that further contributed to the recovery of synaptic density in hippocampus [75]. Although the transplantation of NSCs can improve cognitive deficits significantly, their contributions to the prevention of AD pathology remain questionable, which may raise the possibility of AD return in long-term observation. To address this issue, Blurton-Jones et al. introduced genetically modified NSCs encoding Aβ-degrading enzyme [80]. The modified NSCs not only increased neural density but also significantly decreased Aβ levels in the hippocampus, as well as surrounding regions [80]. In summary, stem cells combined with such modifications could offer combined therapeutic effects of both supplement of neuronal cells and neurotrophic factors, greatly enhancing the power of stem cell therapy in AD treatment.

#### 4.2.3. Challenges

Although various studies have shown promising therapeutic effects of stem cell transplantation, there are some challenges that need to be taken into consideration. First, the prolonged therapeutic effects in stem cell transplantation remain unclear. To the best of the authors’ knowledge, the longest observation period of behavior and cognition improvement after transplantation into mice models were 4 months [266]. It has been reported that the transplanted stem cells were found to decrease over the time in the host microenvironment [270,271], which may decrease the therapeutic effects of engrafted stem cells in the long-term treatment. Second, the possibility of tumor formation after stem cell transplantation is another major obstacle to pass clinical trials [272,273]. A number of studies revealed that stem cells were possessing features found in cancer stem cells, such as obtaining increased apoptosis resistance and prolonged life span [272]. These challenges suggest more studies on the investigation of long-term effects and side effects during cell-based neuroregeneration therapy.

We summarized all medicines mentioned above in Table 1.

## 5. Outlook

AD is a progressive disease devastating brain functions, such as memory, language, and social behavior, as well as even threatening the life for millions of people in the world as no definite cure exists. In this review, we highlighted the current and future treatment options targeting each stage of AD progression. The major characteristics observed in an early AD stage involve the accumulation of Aβ and tau proteins in the brains [12,82]. Conventional treatment options targeting the Aβ plaques and tau tangles are merely relieving symptoms for mild and moderate AD patients, which are not able to alleviate brain damages caused by oxidative stress and neuroinflammation, frequently observed in a severe AD stage [109,126]. To develop a novel therapeutic option to cure further severe AD, enormous efforts have been delivered and suggested to adopt strategies of antioxidation, anti-inflammation, or neuroregeneration. Despite promising outcomes from above mentioned strategies tested in in vitro and in vivo models, untargeted methods caused significant side effects in the clinical trials [150]. In addition, the presence of BBB blocked the entrance of most therapeutics (chemo-, antibody-, or cell-based modules) to CNS representing that further delivery method passing through BBB is urgently in needed [75,159,264]. Therefore, future studies on the underlying mechanisms of AD progression from early to late stages in both molecular and cellular levels, as well as the targeted delivery methods for the AD therapeutics, should be performed to discover novel therapeutic cures for AD.

## Figures and Tables

**Table 1:** List of drugs for the current treatment or potent treatment for Alzheimer’s Disease.

Class of Drugs	Compound	Mechanisms	Status	Details *	[Ref]
1. Targeting Aβ pathology					
Inhibition of Aβ cascade	Verubecestat	β-secretase inhibitor	Phase II/III, failed	1958/55.3/71.8	[18]
	Lanabecestat	β-secretase inhibitor	Phase II/III, failed	2218/53.1/71.3	[19]
	Elenbecestat	β-secretase inhibitor	Phase III	Not recruiting yet	[20]
	Atabecestat	β-secretase inhibitor	Phase II/III, failed	63/54.8/69.1	[21]
	CNP520	β-secretase inhibitor	Phase III, failed	1145/NA/NA	[22]
	Tarenflurbil	γ -secretase inhibitor	Phase III, failed	1649/50.9/74.6	[23]
	Semagacestat	γ -secretase inhibitor	Phase II, failed	1534/53.0/73.2	[24]
Passive immunotherapy	Bapineuzumab	Clearance of oligomeric Aβ	Phase II/III, failed	2204/53.9/72.4	[25]
	Gantenerumab	Clearance of oligomeric Aβ	Phase II/III, failed	797/NA/70.3	[26]
Inhibition of Aβ aggregation	PBT2	Anti-Aβ aggregation	Phase II	78/86.7/71.9	[27]
2. Targeting tauopathy					
Tau expression inhibitors	Antisense nucleotides	Reducing tau mRNA and protein	In vitro	PS19 mice with Tau/NA	[28]
Phosphatase modifiers	Okadaic acid	PP2A inhibitor	In vitro & in vivo	Sparague-Dawley rats/Male	[29]
	Calyculin A	PP2A inhibitor	In vitro & in vivo	Sparague-Dawley rats/NA	[30]
	Memantine	PP2A inhibitor	In vitro	PC12 cells	[31]
	SEW2871	PP2A activiator	In vivo	Sparague-Dawley rats/Male	[32]
	Zinc Chelators	PP2A activiator	In vivo	Sparague-Dawley rats/Male	[33]
Tau kinase inhibitors	Tideglusib	GSK3-β inhibitor	Phase II	306/55.5/71.5	[34]
	Lithium	GSK3-β inhibitor	Phase II	71/NA/NA	[35]
	Amino-thiazole	GSK3-β inhibitor	In vitro	Sf21 cells	[36]
	Thiadiazolidinone	GSK3-β inhibitor	In vitro	Kinase assay	[37]
	Sirenade	GSK3-β inhibitor	In vivo	Wistar rats/Male	[38]
	R-roscovitine	CDK inhibitor	In vivo	Wistar rats/Both	[39]
	Aloisine	CDK inhibitor	In vitro	NT2 cells	[40]
Inhibition of tau acetylation	Salsalate	Tau acetylation inhibitor	In vivo	PS19 mice/NA	[41]
Tau glycosylation	p-diesterase IV inhibitor	cAMP activator	Phase III	336/56.8/74.0	[42]
Tau aggregation inhibitors	LMTX	Dissolving tau filaments	Phase III	885/62.0/70.6	[43]
	Rhodamine	Dissolving tau oligomer	In vitro & in vivo	Neurons from PC12 cells	[44]
	N-Phenylamines	Inhibition of tau aggregation	In vitro	N2a cells expressing tau	[45]
	RemberTM	Dissolving aggregates	Phase II	321/54.0/73.8	[46]
Microtubule stabilizers	Epothilone D	Increasing axonal MT density	In vivo	PS19 mice/Male	[47]
	NAP	Tubulin stabilizer	In vitro & in vivo	Motor neurons with Tau mutant	[48]
	TPI 287	Tubulin stabilizer	Phase I	26/54.0/63.0	[49]
3. Modulation of neuroinflammation					
Regulation of innate immunity	Stattic	JAK2/STAT3 inhibitor	In vivo	5XFAD mice	[50]
	SB203580	p38 MAPK inhibitor	In vitro	Cortical neuron	[51]
	SB202190	p38 MAPK inhibitor	In vivo	Rats with vascular dementia/NA	[52]
	SP600125	p38 MAPK inhibitor	In vivo	Mice with traumatic injury/NA	[53]
	NJK14047	p38 MAPK inhibitor	In vitro & in vivo	BV2 cells & Mice with LPS/NA	[54]
	VIVIT peptide	Calcineurin/NFAT inhibitor	In vivo model	APP/PS1 mice/NA	[55]
	Tacrolimus	Calcineurin/NFAT inhibitor	Phase II	Not recruiting yet	[56]
	Anti-apoE	TREM2 activator	In vivo	APP/PS1 mice/Male	[57]
	Anti-TREM2	TREM2 activator	In vivo	Mice with TREM2 mutant/NA	[58]
	GW2580	CSF1R inhibitor	In vivo	APP, PSEN, APP/PS1 mice/NA	[59]
	MRS2179	P2Y1R antagonists	In vivo	APP/PS1 mice/NA	[60]
Regulation of adaptive immunity	Aβ-specific Th2 lymphocytes	Regulation of adaptive immunity	In vivo	APP/PS1 mice/NA	[61]
	anti-Aβ IgG	Neutralization of fibrinogenesis	In vivo	5XFAD mice/NA	[62]
4. Neuroregeneration Restoring Cognitive Impairment					
Supplement of neurotrophins	BDNF	Neurotrophic factors	In vivo	Rats with Aβ1–42/NA	[63]
	hNGFp	Neurotrophic factors	In vivo	APP/PS1 mice/NA	[64]
	AAV2-NGF	Neurotrophic factors	Phase II	49/43.0/68.0	[65]
Neurotrophin mimetics	7,8-DHF	BDNF mimetic	In vivo	5XFAD mice/NA	[66]
	Doxygedunin	BDNF mimetic	In vivo	Mice with TrkB mutant/NA	[67]
	LM22A-4	BDNF mimetic	In vitro & in vivo	Mice with TrkB mutant/Male	[68]
	Bicyclic BDNF loop mimetic	BDNF mimetic	In vitro	Primary sensory neurons	[69]
Conversion of glial cells to neuronal cells	Neurogenin 2	Astrocytes to glutamatergic neurons	In vitro	Postnatal cortical astroglia	[70]
	Dlx2	Astrocytes to GABAergic neurons	In vitro	Postnatal cortical astroglia	[71]
	Combination of 9 molecules	Astrocytes to neurons	In vitro & in vivo	Astrocytes	[72]
	Combination of 4 molecules	Astrocytes to neurons	In vitro & in vivo	Cortical astrocytes	[73]
Transplanting stem cells into brains	NSCs encoding with NGF	NSCs to neurons and astrocytes	In vivo	Mice with dementia/NA	[74]
	NSCs encoding with BDNF	NSCs to neurons	In vivo	APP mice/NA	[75]
	NSCs pretreated with BDNF	NSCs to cholinergic neurons	In vivo	APP/PS1 mice/NA	[76]
	NSCs expressing IGF-I	Engraftment of HK532-IGF-I cells	In vitro & in vivo	APP/PS1 mice/NA	[77]
	BM-MSCs expressing VEGF	Decrease in senile plaque	In vivo	APP/PS1 mice/NA	[78]
	MSCs expressing GLP-1	Decrease in Aβ plaque deposition	In vivo	APP mice/Male	[79]
	NSCs expressing neprilysin	Decrease in Aβ pathology	In vivo	APP mice/NA	[80]

* Clinical studies are represented as Case number/Female percentage/Mean Age; in vitro studies are represented as Cellular models or Assays; in vivo studies are represented as Name of the animal model/Sex. NA represents Not Available.

## Abbreviations

7,8-DHF	7,8-dihydroxyflavone
AAV2	Adeno-associated virus serotype 2
AD	Alzheimer’s Disease
ALS	Amyotrophic lateral sclerosis
APOE	Apolipoprotein E
APP	Amyloid-beta precursor protein
ATP	Adenosine Triphosphate
Aβ	Amyloid beta
APLP1	Amyloid-like protein 1
BAC	Bacterial Artificial Chromosome
BACE-1	Beta-site APP Cleaving Enzyme 1
BDNF	Brain-Derived Neurotrophic Factor
CAMK II	Ca2^+^/calmodulin-dependent protein kinase II
CAT	Catalase
CBD	Corticobasal Degeneration
CDK5	Cycle-dependent Kinase 5
CHL1	Close homolog of L1
CNS	Central Nervous System
CNTFs	Ciliary Neurotrophic Factors
COX	Cyclooxygenase
CSF	Cerebrospinal Fluid
CSF1R	Colony-Stimulating Factor-1 Receptor
DAM	Disease-Associated Microglia
DAPT	N-(N-(3,5-difluorophenacetyl)-L-alanyl)-S-phenylglycine t-butyl ester
DNA	Deoxyribonucleic acid
ERK	Extracellular signal Regulated Kinase
FAD	Familial Alzheimer’s Disease
FTDP-17	Frontotemporal dementia with parkinsonism-17
GDNFs	Glial cell line-Derived Neurotrophic Factors
GABAergic	γ-Aminobutyric Acid-producing
GFAP	Glial Fibrillary Acidic Protein
GLP-1	Glucagon-Like Peptide-1
GPX	Glutathione Peroxidase
GSH	Glutathione
GSK-3β	Glycogen synthase kinase 3 beta
hNGFp	painless NGF protein
IFN-γ	Interferon gamma
IFITM3	Interferon-induced transmembrane protein 3
IGF-I	Insulin-like Growth Factor-I
IgG	Immunoglobulin G
IL	Interleukin
JAK	Janus Kinase
LMTM	Leuco-methylthioninium bis (hydro-methanesulfonate)
MAPK	Mitogen-Activated Protein Kinase
MB	Methylene blue
MCI	Mild cognitive impairment
MPACs	Metal protein attenuating compounds
MSCs	Mesenchymal Stem Cells
NADP	Nicotinamide adenine dinucleotide phosphate
NRF2	Nuclear factor erythroid 2-related factor
NFAT	Nuclear Factor of Activated T-cells
NFT	Neurofibrillary Tangles
NF-κB	Nuclear factor-κB
NGF	Nerve Growth Factor
NLR	Nod-Like Receptor
NLRP3	NLR family Pyrin domain containing 3
NO	Nitric Oxide
NSAIDs	Non-Steroidal Anti-Inflammatory Drugs
NSCs	Neural Stem Cells
NT	Neurotrophin
OKA	Okadaic acid
P2Y1R	P2Y1 purinoreceptor
PET	Positron emission tomography
PD	Parkinson’s Disease
PHF	Paired Helical Filament
PI3K	Phosphatidylinositol 3-Kinase
PiD	Pick Disease
PKA	cAMP-dependent protein kinase
PP	Protein phosphatase
PPZ	Perphenazine
PS	Presenilin
PSP	Progressive Supranuclear Palsy
RAGE	Receptor for Advanced Glycosylation End products
RNA	Ribonucleic acid
RNS	Reactive Nitrogen Species
ROS	Reactive Oxygen Species
SAD	Sporadic Alzheimer’s Disease
SAG	Smoothened Agonist
SOCS3	Suppressor of Cytokine Signaling 3
SOD	Superoxide Dismutase
STAT	Signal Transducer and Activator of Transcription
sTREM2	Soluble TREM2
TBB	4,5,6,7-tetrabromobenzotriazole
TGF-β	Transforming growth factor beta
Th1	Type I helper T cells
Th2	Type II helper T cells
TLR	Toll-Like Receptor
TNF-α	Tumor Necrosis Factor
Tregs	Regulation T cells
TREM2	Triggering Receptor Expressed on Myeloid cells 2
Trks	Tropomyosin Receptor Kinases
TTNPB	4-((E)-2-(5,6,7,8-Tetrahydro-5,5,8,8-tetramethyl-2-naphthalenyl)-1-propenyl)-benzoic acid
VEGF	Vascular Endothelial Growth Factor
VPA	Valproic Acid

## References

[1] Ferri C.P., Prince M., Brayne C., Brodaty H., Fratiglioni L., Ganguli M., Hall K., Hasegawa K., Hendrie H., Huang Y. (2005). Global Prevalence of Dementia: A Delphi Consensus Study. Lancet.

[2] Patterson C. (2018). World Alzheimer Report 2018—The State of the Art of Dementia Research: New Frontiers.

[3] Martin P., Anders W., Maëlenn G., Ali G., Yu-Tzu W., Matthew P. (2015). World Alzheimer Report 2015: The Global Impact of Dementia—An Analysis of Prevalence, Incidence, Cost and Trends.

[4] Ledig C., Schuh A., Guerrero R., Heckemann R.A., Rueckert D. (2018). Structural Brain Imaging in Alzheimer’s Disease and Mild Cognitive Impairment: Biomarker Analysis and Shared Morphometry Database. Sci. Rep..

[5] Alzheimer’s Association (2019). 2019 Alzheimer’s Disease Facts and Figures. Alzheimer’s Dement..

[6] Mensah G.A., Wei G.S., Sorlie P.D., Fine L.J., Rosenberg Y., Kaufmann P.G., Mussolino M.E., Hsu L.L., Addou E., Engelgau M.M. (2017). Decline in Cardiovascular Mortality: Possible Causes and Implications. Circ. Res..

[7] Siegel R.L., Miller K.D., Jemal A. (2020). Cancer Statistics, 2020. Cancer J. Clin..

[8] Lackland D.T., Roccella E.J., Deutsch A.F., Fornage M., George M.G., Howard G., Kissela B.M., Kittner S.J., Lichtman J.H., Lisabeth L.D. (2014). Factors Influencing the Decline in Stroke Mortality a Statement from the American Heart Association/American Stroke Association. Stroke.

[9] WHO Dementia. https://www.who.int/news-room/fact-sheets/detail/dementia.

[10] Murphy M.P., LeVine H. (2010). Alzheimer’s Disease and the β-Amyloid Peptide. J. Alzheimer’s Dis..

[11] Hardy J., Allsop D. (1991). Amyloid Deposition as the Central Event in the Aetiology of Alzheimer’s Disease. Trends Pharmacol. Sci..

[12] Espíndola S.L., Damianich A., Alvarez R.J., Sartor M., Belforte J.E., Ferrario J.E., Gallo J.M., Avale M.E. (2018). Modulation of Tau Isoforms Imbalance Precludes Tau Pathology and Cognitive Decline in a Mouse Model of Tauopathy. Cell Rep..

[13] Kosik K.S., Joachim C.L., Selkoe D.J. (1986). Microtubule-Associated Protein Tau (τ) Is a Major Antigenic Component of Paired Helical Filaments in Alzheimer Disease. Proc. Natl. Acad. Sci. USA.

[14] Houck A.L., Hernández F., Ávila J. (2016). A Simple Model to Study Tau Pathology. J. Exp. Neurosci..

[15] Kinney J.W., Bemiller S.M., Murtishaw A.S., Leisgang A.M., Salazar A.M., Lamb B.T. (2018). Inflammation as a Central Mechanism in Alzheimer’s Disease. Alzheimer’s Dement. Transl. Res. Clin. Interv..

[16] Gaudet A.D., Fonken L.K. (2018). Glial Cells Shape Pathology and Repair After Spinal Cord Injury. Neurotherapeutics.

[17] Sawikr Y., Yarla N.S., Peluso I., Kamal M.A., Aliev G., Bishayee A. (2017). Neuroinflammation in Alzheimer’s Disease: The Preventive and Therapeutic Potential of Polyphenolic Nutraceuticals. Adv. Protein Chem. Struct. Biol..

[18] Egan M.F., Kost J., Tariot P.N., Aisen P.S., Cummings J.L., Vellas B., Sur C., Mukai Y., Voss T., Furtek C. (2018). Randomized Trial of Verubecestat for Mild-to-Moderate Alzheimer’s Disease. N. Engl. J. Med..

[19] Malone E. Lilly/AstraZeneca’s Lanabecestat Becomes Latest BACE Inhibitor Casualty. https://scrip.pharmaintelligence.informa.com/SC123243/LillyAstraZenecas-Lanabecestat-BecomesLatest-BACE-Inhibitor-Casualty.

[20] Hung S.Y., Fu W.M. (2017). Drug Candidates in Clinical Trials for Alzheimer’s Disease. J. Biomed. Sci..

[21] Update on Janssen’s BACE Inhibitor Program Regarding the Dominantly Inherited Alzheimer’s Network Trial (DIAN-TU). https://www.janssen.com/neuroscience/update-janssens-bace-inhibitor-program-regarding-DIAN-TU.

[22] Doody R.S., Raman R., Farlow M., Iwatsubo T., Vellas B., Joffe S., Kieburtz K., He F., Sun X., Thomas R.G. (2013). A Phase 3 Trial of Semagacestat for Treatment of Alzheimer’s Disease. N. Engl. J. Med..

[23] Green R.C., Schneider L.S., Amato D.A., Beelen A.P., Wilcock G., Swabb E.A., Zavitz K.H. (2009). Effect of Tarenflurbil on Cognitive Decline and Activities of Daily Living in Patients with Mild Alzheimer Disease: A Randomized Controlled Trial. JAMA.

[24] Coric V., Van Dyck C.H., Salloway S., Andreasen N., Brody M., Richter R.W., Soininen H., Thein S., Shiovitz T., Pilcher G. (2012). Safety and Tolerability of the γ-Secretase Inhibitor Avagacestat in a Phase 2 Study of Mild to Moderate Alzheimer Disease. Arch. Neurol..

[25] Khorassani F., Hilas O. (2013). Bapineuzumab, an Investigational Agent for Alzheimer’s Disease. Pharm. Ther..

[26] Ostrowitzki S., Lasser R.A., Dorflinger E., Scheltens P., Barkhof F., Nikolcheva T., Ashford E., Retout S., Hofmann C., Delmar P. (2017). A Phase III Randomized Trial of Gantenerumab in Prodromal Alzheimer’s Disease. Alzheimer’s Res. Ther..

[27] Lannfelt L., Blennow K., Zetterberg H., Batsman S., Ames D., Harrison J., Masters C.L., Targum S., Bush A.I., Murdoch R. (2008). Safety, Efficacy, and Biomarker Findings of PBT2 in Targeting Aβ as a Modifying Therapy for Alzheimer’s Disease: A Phase IIa, Double-Blind, Randomised, Placebo-Controlled Trial. Lancet Neurol..

[28] DeVos S.L., Miller R.L., Schoch K.M., Holmes B.B., Kebodeaux C.S., Wegener A.J., Chen G., Shen T., Tran H., Nichols B. (2017). Tau Reduction Prevents Neuronal Loss and Reverses Pathological Tau Deposition and Seeding in Mice with Tauopathy. Sci. Transl. Med..

[29] Kamat P.K., Rai S., Nath C. (2013). Okadaic Acid Induced Neurotoxicity: An Emerging Tool to Study Alzheimer’s Disease Pathology. Neurotoxicology.

[30] Yang X., Yang Y., Fu Z., Li Y., Feng J., Luo J., Zhang Q., Wang Q., Tian Q. (2011). Melatonin Ameliorates Alzheimer-like Pathological Changes and Spatial Memory Retention Impairment Induced by Calyculin A. J. Psychopharmacol..

[31] Chohan M.O., Khatoon S., Iqbal I.G., Iqbal K. (2006). Involvement of I2PP2A in the Abnormal Hyperphosphorylation of Tau and Its Reversal by Memantine. FEBS Lett..

[32] Giguère F.S.-C., Essis S.A., Chagniel L., Germain M., Cyr M., Massicotte G. (2017). The Sphingosine-1-Phosphate Receptor 1 Agonist SEW2871 Reduces Tau-Ser262 Phosphorylation in Rat Hippocampal Slices. Brain Res..

[33] Xiong Y., Jing X.P., Zhou X.W., Wang X.L., Yang Y., Sun X.Y., Qiu M., Cao F.Y., Lu Y.M., Liu R. (2013). Zinc Induces Protein Phosphatase 2A Inactivation and Tau Hyperphosphorylation through Src Dependent PP2A (Tyrosine 307) Phosphorylation. Neurobiol. Aging.

[34] Lovestone S., Boada M., Dubois B., Hüll M., Rinne J.O., Huppertz H.J., Calero M., Andrés M.V., Gómez-Carrillo B., León T. (2015). A Phase II Trial of Tideglusib in Alzheimer’s Disease. J. Alzheimer’s Dis..

[35] Harald H., Michael E., Katharina B., Peter A., Anette M., Anna B., Lutz F., Johannes S., Peter S., Matthias W.R. (2009). Lithium Trial in Alzheimer’s Disease: A Randomized, Single-Blind, Placebo-Controlled, Multicenter 10-Week Study. J. Clin. Psychiatry.

[36] Ratan B., Yafeng X., Stefan B., Sven H., Mats O., Yvonne N., Ann-Cathrin R., Eva J., Per-Olof M., Thomas B. (2003). Structural Insights and Biological Effects of Glycogen Synthase Kinase 3-Specific Inhibitor AR-A014418. J. Biol. Chem..

[37] Martinez A., Alonso M., Castro A., Pérez C., Moreno F.J. (2002). First Non-ATP Competitive Glycogen Synthase Kinase 3 β (GSK-3β) Inhibitors: Thiadiazolidinones (TDZD) as Potential Drugs for the Treatment of Alzheimer’s Disease. J. Med. Chem..

[38] Le Corre S., Klafki H.W., Plesnila N., Hübinger G., Obermeier A., Sahagún H., Monse B., Seneci P., Lewis J., Eriksen J. (2006). An Inhibitor of Tau Hyperphosphorylation Prevents Severe Motor Impairments in Tau Transgenic Mice. Proc. Natl. Acad. Sci. USA.

[39] Ivanov A., Tyzio R., Zilberter Y., Ben-Ari Y. (2008). (R)-Roscovitine, a Cyclin-Dependent Kinase Inhibitor, Enhances Tonic GABA Inhibition in Rat Hippocampus. Neuroscience.

[40] Mettey Y., Gompel M., Thomas V., Garnier M., Leost M., Ceballos-Picot I., Noble M., Endicott J., Vierfond J.M., Meijer L. (2003). Aloisines, a New Family of CDK/GSK-3 Inhibitors. SAR Study, Crystal Structure in Complex with CDK2, Enzyme Selectivity, and Cellular Effects. J. Med. Chem..

[41] Min S.W., Chen X., Tracy T.E., Li Y., Zhou Y., Wang C., Shirakawa K., Minami S.S., Defensor E., Mok S.A. (2015). Critical Role of Acetylation in Tau-Mediated Neurodegeneration and Cognitive Deficits. Nat. Med..

[42] Sanders O., Rajagopal L. (2020). Phosphodiesterase Inhibitors for Alzheimer’s Disease: A Systematic Review of Clinical Trials and Epidemiology with a Mechanistic Rationale. J. Alzheimer’s Dis. Rep..

[43] Gauthier S., Feldman H.H., Schneider L.S., Wilcock G.K., Frisoni G.B., Hardlund J.H., Moebius H.J., Bentham P., Kook K.A., Wischik D.J. (2016). Efficacy and Safety of Tau-Aggregation Inhibitor Therapy in Patients with Mild or Moderate Alzheimer’s Disease: A Randomised, Controlled, Double-Blind, Parallel-Arm, Phase 3 Trial. Lancet.

[44] Pradhan K., Das G., Kar C., Mukherjee N., Khan J., Mahata T., Barman S., Ghosh S. (2020). Rhodamine-Based Metal Chelator: A Potent Inhibitor of Metal-Catalyzed Amyloid Toxicity. ACS Omega.

[45] Pickhardt M., Biernat J., Khlistunova I., Wang Y.-P., Gazova Z., Mandelkow E.-M., Mandelkow E. (2007). N-Phenylamine Derivatives as Aggregation Inhibitors in Cell Models of Tauopathy. Curr. Alzheimer Res..

[46] Wischik C.M., Staff R.T., Wischik D.J., Bentham P., Murray A.D., Storey J.M.D., Kook K.A., Harrington C.R. (2015). Tau Aggregation Inhibitor Therapy: An Exploratory Phase 2 Study in Mild or Moderate Alzheimer’s Disease. J. Alzheimer’s Dis..

[47] Zhang B., Carroll J., Trojanowski J.Q., Yao Y., Iba M., Potuzak J.S., Hogan A.M.L., Xie S.X., Ballatore C., Smith A.B. (2012). The Microtubule-Stabilizing Agent, Epothilone D, Reduces Axonal Dysfunction, Neurotoxicity, Cognitive Deficits, and Alzheimer-like Pathology in an Interventional Study with Aged Tau Transgenic Mice. J. Neurosci..

[48] Quraishe S., Cowan C.M., Mudher A. (2013). NAP (Davunetide) Rescues Neuronal Dysfunction in a Drosophila Model of Tauopathy. Mol. Psychiatry.

[49] Tsai R.M., Miller Z., Koestler M., Rojas J.C., Ljubenkov P.A., Rosen H.J., Rabinovici G.D., Fagan A.M., Cobigo Y., Brown J.A. (2020). Reactions to Multiple Ascending Doses of the Microtubule Stabilizer TPI-287 in Patients with Alzheimer Disease, Progressive Supranuclear Palsy, and Corticobasal Syndrome: A Randomized Clinical Trial. JAMA Neurol..

[50] Choi M., Kim H., Yang E.J., Kim H.S. (2020). Inhibition of STAT3 Phosphorylation Attenuates Impairments in Learning and Memory in 5XFAD Mice, an Animal Model of Alzheimer’s Disease. J. Pharmacol. Sci..

[51] Liu X.W., Ji E.F., He P., Xing R.X., Tian B.X., Li X.D. (2014). Protective Effects of the P38 MAPK Inhibitor SB203580 on NMDA-Induced Injury in Primary Cerebral Cortical Neurons. Mol. Med. Rep..

[52] Yang S., Zhou G., Liu H., Zhang B., Li J., Cui R., Du Y. (2013). Protective Effects of P38 MAPK Inhibitor SB202190 against Hippocampal Apoptosis and Spatial Learning and Memory Deficits in a Rat Model of Vascular Dementia. BioMed Res. Int..

[53] Rehman S.U., Ahmad A., Yoon G.H., Khan M., Abid M.N., Kim M.O. (2018). Inhibition of C-Jun N-Terminal Kinase Protects against Brain Damage and Improves Learning and Memory after Traumatic Brain Injury in Adult Mice. Cereb. Cortex.

[54] Gee M.S., Kim S.W., Kim N., Lee S.J., Oh M.S., Jin H.K., Bae J.S., Inn K.S., Kim N.J., Lee J.K. (2018). A Novel and Selective P38 Mitogen-Activated Protein Kinase Inhibitor Attenuates LPS-Induced Neuroinflammation in BV2 Microglia and a Mouse Model. Neurochem. Res..

[55] Rojanathammanee L., Floden A.M., Manocha G.D., Combs C.K. (2015). Attenuation of Microglial Activation in a Mouse Model of Alzheimer’s Disease via NFAT Inhibition. J. Neuroinflamm..

[56] Massachusetts General Hospital (2020). A Pilot Open Labeled Study of Tacrolimus in Alzheimer’s Disease. https://clinicaltrials.gov/ct2/show/NCT04263519.

[57] Kim J., Eltorai A.E.M., Jiang H., Liao F., Verghese P.B., Kim J., Stewart F.R., Basak J.M., Holtzman D.M. (2012). Anti-ApoE Immunotherapy Inhibits Amyloid Accumulation in a Transgenic Mouse Model of Aβ Amyloidosis. J. Exp. Med..

[58] Cheng Q., Danao J., Talreja S., Wen P., Yin J., Sun N., Li C.M., Chui D., Tran D., Koirala S. (2018). TREM2-Activating Antibodies Abrogate the Negative Pleiotropic Effects of the Alzheimer’s Disease Variant Trem2R47H on Murine Myeloid Cell Function. J. Biol. Chem..

[59] Olmos-Alonso A., Schetters S.T.T., Sri S., Askew K., Mancuso R., Vargas-Caballero M., Holscher C., Perry V.H., Gomez-Nicola D. (2016). Pharmacological Targeting of CSF1R Inhibits Microglial Proliferation and Prevents the Progression of Alzheimer’s-like Pathology. Brain.

[60] Reichenbach N., Delekate A., Breithausen B., Keppler K., Poll S., Schulte T., Peter J., Plescher M., Hansen J.N., Blank N. (2018). P2Y1 Receptor Blockade Normalizes Network Dysfunction and Cognition in an Alzheimer’s Disease Model. J. Exp. Med..

[61] Cao C., Arendash G.W., Dickson A., Mamcarz M.B., Lin X., Ethell D.W. (2009). Aβ-Specific Th2 Cells Provide Cognitive and Pathological Benefits to Alzheimer’s Mice without Infiltrating the CNS Chuanhai. Neurobiol. Dis..

[62] Marsh S.E., Abud E.M., Lakatos A., Karimzadeh A., Yeung S.T., Davtyan H., Fote G.M., Lau L., Weinger J.G., Lane T.E. (2016). The Adaptive Immune System Restrains Alzheimer’s Disease Pathogenesis by Modulating Microglial Function. Proc. Natl. Acad. Sci. USA.

[63] Zhang L., Fang Y., Lian Y., Chen Y., Wu T., Zheng Y., Zong H., Sun L., Zhang R., Wang Z. (2015). Brain-Derived Neurotrophic Factor Ameliorates Learning Deficits in a Rat Model of Alzheimer’s Disease Induced by Aβ1-42. PLoS ONE.

[64] Capsoni S., Marinelli S., Ceci M., Vignone D., Amato G., Malerba F., Paoletti F., Meli G., Viegi A., Pavone F. (2012). Intranasal “Painless” Human Nerve Growth Factors Slows Amyloid Neurodegeneration and Prevents Memory Deficits in App × PS1 Mice. PLoS ONE.

[65] Rissman R.A., Siffert J., Aisen P.S., Team A.S. (2018). Adeno-Associated Viral Vector (Serotype 2)—Nerve Growth Factor for Patients with Alzheimer Disease A Randomized Clinical Trial. JAMA Neurol..

[66] Devi L., Ohno M. (2012). 7,8-Dihydroxyflavone, a Small-Molecule TrkB Agonist, Reverses Memory Deficits and BACE1 Elevation in a Mouse Model of Alzheimer’s Disease. Neuropsychopharmacology.

[67] Jang S.W., Liu X., Chan C.B., France S.A., Sayeed I., Tang W., Lin X., Xiao G., Andero R., Chang Q. (2010). Deoxygedunin, a Natural Product with Potent Neurotrophic Activity in Mice. PLoS ONE.

[68] Massa S.M., Yang T., Xie Y., Shi J., Bilgen M., Joyce J.N., Nehama D., Rajadas J., Longo F.M. (2010). Small Molecule BDNF Mimetics Activate TrkB Signaling and Prevent Neuronal Degeneration in Rodents. J. Clin. Investig..

[69] Fletcher J.M., Morton C.J., Zwar R.A., Murray S.S., O’Leary P.D., Hughes R.A. (2008). Design of a Conformationally Defined and Proteolytically Stable Circular Mimetic of Brain-Derived Neurotrophic Factor. J. Biol. Chem..

[70] Heinrich C., Blum R., Gascón S., Masserdotti G., Tripathi P., Sánchez R., Tiedt S., Schroeder T., Götz M., Berninger B. (2010). Directing Astroglia from the Cerebral Cortex into Subtype Specific Functional Neurons. PLoS Biol..

[71] Liu M.H., Li W., Zheng J.J., Xu Y.G., He Q., Chen G. (2020). Differential Neuronal Reprogramming Induced by NeuroD1 from Astrocytes in Grey Matter versus White Matter. Neural Regen. Res..

[72] Zhang L., Yin J.C., Yeh H., Ma N.X., Lee G., Chen X.A., Wang Y., Lin L., Chen L., Jin P. (2015). Small Molecules Efficiently Reprogram Human Astroglial Cells into Functional Neurons. Cell Stem Cell.

[73] Yin J.C., Zhang L., Ma N.X., Wang Y., Lee G., Hou X.Y., Lei Z.F., Zhang F.Y., Dong F.P., Wu G.Y. (2019). Chemical Conversion of Human Fetal Astrocytes into Neurons through Modulation of Multiple Signaling Pathways. Stem Cell Rep..

[74] Lee H.J., Lim I.J., Park S.W., Kim Y.B., Ko Y., Kim S.U. (2012). Human Neural Stem Cells Genetically Modified to Express Human Nerve Growth Factor (NGF) Gene Restore Cognition in the Mouse With Ibotenic Acid-Induced Cognitive Dysfunction. Cell Transplant..

[75] Wu C.C., Lien C.C., Hou W.H., Chiang P.M., Tsai K.J. (2016). Gain of BDNF Function in Engrafted Neural Stem Cells Promotes the Therapeutic Potential for Alzheimer’s Disease. Sci. Rep..

[76] Li T., Yu Y., Cai H. (2015). Effects of Brain-Derived Neurotrophic Factor-Pretreated Neuron Stem Cell Transplantation on Alzheimer’s Disease Model Mice. Int. J. Clin. Exp. Med..

[77] Alfred A. (2016). Human Cortical Neural Stem Cells Expressing Insulin-Like Growth Factor-I: A Novel Cellular Therapy for Alzheimer’s Disease. Stem Cells Transl. Med..

[78] Garcia K.O., Ornellas F.L.M., Martin P.K.M., Patti C.L., Mello L.E., Frussa-filho R., Han S.W., Longo B.M. (2014). Therapeutic Effects of the Transplantation of VEGF Overexpressing Bone Marrow Mesenchymal Stem Cells in the Hippocampus of Murine Model of Alzheimer’ s Disease. Front. Aging Neurosci..

[79] Klinge P.M., Harmening K., Miller M.C., Heile A., Wallrapp C., Geigle P., Brinker T. (2011). Encapsulated Native and Glucagon-like Peptide-1 Transfected Human Mesenchymal Stem Cells in a Transgenic Mouse Model of Alzheimer’s Disease. Neurosci. Lett..

[80] Blurton-Jones M., Spencer B., Michael S., Castello N.A., Agazaryan A.A., Davis J.L., Müller F., Loring J.F., Masliah E., LaFerla F.M. (2014). Neural Stem Cells Genetically-Modified to Express Neprilysin Reduce Pathology in Alzheimer Transgenic Models. Stem Cell Res. Ther..

[81] Zhang X., Fu Z., Meng L., He M., Zhang Z. (2018). The Early Events That Initiate β-Amyloid Aggregation in Alzheimer’s Disease. Front. Aging Neurosci..

[82] LaFerla F.M., Green K.N., Oddo S. (2007). Intracellular Amyloid-β in Alzheimer’s Disease. Nat. Rev. Neurosci..

[83] Zhang C., Tanzi R.E. (2012). Natural Modulators of Amyloid-Beta Precursor Protein Processing. Curr. Alzheimer Res..

[84] O’Brein R.J., Wong P.C. (2011). Amyloid Precursor Protein Processing and Alzheimer’s Disease. Annu. Rev. Neurosci..

[85] Chen G.-F., Xu T.-H., Yan Y., Zhou Y.-R., Jiang Y., Melcher K., Xu H.E. (2017). Amyloid Beta: Structure, Biology and Structure-Based Therapeutic Development. Acta Pharmacol. Sin..

[86] Citron M., Diehl T.S., Gordon G., Biere A.L., Seubert P., Selkoe D.J. (1996). Evidence That the 42- and 40-Amino Acid Forms of Amyloid β Protein Are Generated from the β-Amyloid Precursor Protein by Different Protease Activities. Proc. Natl. Acad. Sci. USA.

[87] Querfurth H.W., LaFerla F.M. (2010). Mechanisms of Disease Alzheimer’s Disease. N. Engl. J. Med..

[88] Zhou L., McInnes J., Wierda K., Holt M., Herrmann A.G., Jackson R.J., Wang Y.C., Swerts J., Beyens J., Miskiewicz K. (2017). Tau Association with Synaptic Vesicles Causes Presynaptic Dysfunction. Nat. Commun..

[89] Navarro-Yepes J., Burns M., Anandhan A., Khalimonchuk O., del Razo L.M., Quintanilla-Vega B., Pappa A., Panayiotidis M.I., Franco R. (2014). Oxidative Stress, Redox Signaling, and Autophagy: Cell Death versus Survival. Antioxid. Redox Signal..

[90] Mattson M.P. (2004). Pathways towards and Away from Alzheimer’s Disease. Nature.

[91] Haroon E., Miller A.H., Sanacora G. (2017). Inflammation, Glutamate, and Glia: A Trio of Trouble in Mood Disorders. Neuropsychopharmacology.

[92] Evans L.D., Wassmer T., Fraser G., Smith J., Perkinton M., Billinton A., Livesey F.J. (2018). Extracellular Monomeric and Aggregated Tau Efficiently Enter Human Neurons through Overlapping but Distinct Pathways. Cell Rep..

[93] Wang J., Dickson D.W., Trojanowski J.Q., Lee V.M.Y. (1999). The Levels of Soluble versus Insoluble Brain Aβ Distinguish Alzheimer’s Disease from Normal and Pathologic Aging. Exp. Neurol..

[94] Weggen S., Beher D. (2012). Molecular Consequences of Amyloid Precursor Protein and Presenilin Mutations Causing Autosomal-Dominant Alzheimer’s Disease. Alzheimer’s Res. Ther..

[95] Noguchi A., Matsumura S., Dezawa M., Tada M., Yanazawa M., Ito A., Akioka M., Kikuchi S., Sato M., Noda M. (2009). Isolation and Characterization of Patient-Derived, Toxic, High Mass Amyloid β-Protein (Aβ) Assembly from Alzheimer Disease Brains. J. Biol. Chem..

[96] Lim Y.Y., Maruff P., Pietrzak R.H., Ellis K.A., Darby D., Ames D., Harrington K., Martins R.N., Masters C.L., Szoeke C. (2014). Aβ and Cognitive Change: Examining the Preclinical and Prodromal Stages of Alzheimer’s Disease. Alzheimer’s Dement..

[97] Klementieva O., Willén K., Martinsson I., Israelsson B., Engdahl A., Cladera J., Uvdal P., Gouras G.K. (2017). Pre-Plaque Conformational Changes in Alzheimer’s Disease-Linked Aβ and APP. Nat. Commun..

[98] Wan W., Cao L., Liu L., Zhang C., Kalionis B., Tai X., Li Y., Xia S. (2015). Aβ1-42 Oligomer-Induced Leakage in an in Vitro Blood-Brain Barrier Model Is Associated with up-Regulation of RAGE and Metalloproteinases, and down-Regulation of Tight Junction Scaffold Proteins. J. Neurochem..

[99] Elvis C., Hector R., Susan M.B., Manue A.R., Aida G., Syed Z., Syed F.A., Sumit S. (2019). Amyloid Beta 25–35 Induces Blood-Brain Barrier Disruption in Vitro. Metab. Brain Dis..

[100] Querol-Vilaseca M., Colom-Cadena M., Pegueroles J., Nuñez-Llaves R., Luque-Cabecerans J., Muñoz-Llahuna L., Andilla J., Belbin O., Spires-Jones T.L., Gelpi E. (2019). Nanoscale Structure of Amyloid-β Plaques in Alzheimer’s Disease. Sci. Rep..

[101] Pickett E.K., Koffie R.M., Wegmann S., Henstridge C.M., Herrmann A.G., Colom-Cadena M., Lleo A., Kay K.R., Vaught M., Soberman R. (2016). Non-Fibrillar Oligomeric Amyloid-β within Synapses. J. Alzheimer’s Dis..

[102] Pickett E.K., Herrmann A.G., McQueen J., Abt K., Dando O., Tulloch J., Jain P., Dunnett S., Sohrabi S., Fjeldstad M.P. (2019). Amyloid Beta and Tau Cooperate to Cause Reversible Behavioral and Transcriptional Deficits in a Model of Alzheimer’s Disease. Cell Rep..

[103] Hur J.Y., Frost G.R., Wu X., Crump C., Pan S.J., Wong E., Barros M., Li T., Nie P., Zhai Y. (2020). The Innate Immunity Protein IFITM3 Modulates γ-Secretase in Alzheimer’s Disease. Nature.

[104] Kametani F., Hasegawa M. (2018). Reconsideration of Amyloid Hypothesis and Tau Hypothesis in Alzheimer’s Disease. Front. Neurosci..

[105] Tai L.M., Bilousova T., Jungbauer L., Roeske S.K., Youmans K.L., Yu C., Poon W.W., Cornwell L.B., Miller C.A., Vinters H.V. (2013). Levels of Soluble Apolipoprotein E/Amyloid-β (Aβ) Complex Are Reduced and Oligomeric Aβ Increased with APOE4 and Alzheimer Disease in a Transgenic Mouse Model and Human Samples. J. Biol. Chem..

[106] Alzheimer’s Association (2017). 2017 Alzheimer’s Disease Facts and Figures. Alzheimer’s Dement..

[107] Holland D., Desikan R.S., Dale A.M., McEvoy L.K. (2012). Rates of Decline in Alzheimer Disease Decrease with Age. PLoS ONE.

[108] Gao Y.-L., Wang N., Sun F.-R., Cao X.-P., Zhang W., Yu J.-T. (2018). Tau in Neurodegenerative Disease. Ann. Transl. Med..

[109] Alonso A.D.C., Zaidi T., Novak M., Grundke-Iqbal I., Iqbal K. (2001). Hyperphosphorylation Induces Self-Assembly of τ into Tangles of Paired Helical Filaments/Straight Filaments. Proc. Natl. Acad. Sci. USA.

[110] Adams S.J., de Ture M.A., McBride M., Dickson D.W., Petrucelli L. (2010). Three Repeat Isoforms of Tau Inhibit Assembly of Four Repeat Tau Filaments. PLoS ONE.

[111] Wang Y., Martinez-Vicente M., Krüger U., Kaushik S., Wong E., Mandelkow E.M., Cuervo A.M., Mandelkow E. (2009). Tau Fragmentation, Aggregation and Clearance: The Dual Role of Lysosomal Processing. Hum. Mol. Genet..

[112] Li X., Lu F., Wang J.Z., Gong C.X. (2006). Concurrent Alterations of *O*-GlcNAcylation and Phosphorylation of Tau in Mouse Brains during Fasting. Eur. J. Neurosci..

[113] Frost B., Jacks R.L., Diamond M.I. (2009). Propagation of Tau Misfolding from the Outside to the inside of a Cell. J. Biol. Chem..

[114] Zhao Y., Tseng I.C., Heyser C.J., Rockenstein E., Mante M., Adame A., Zheng Q., Huang T., Wang X., Arslan P.E. (2015). Appoptosin-Mediated Caspase Cleavage of Tau Contributes to Progressive Supranuclear Palsy Pathogenesis. Neuron.

[115] Bardai F.H., Wang L., Mutreja Y., Yenjerla M., Gamblin T.C., Feany M.B. (2018). A Conserved Cytoskeletal Signaling Cascade Mediates Neurotoxicity of FTDP-17 Tau Mutations in Vivo. J. Neurosci..

[116] Nelson P.T., Alafuzoff I., Bigio E.H., Bouras C., Braak H., Cairns N.J., Castellani R.J., Crain B.J., Davies P., del Tredici K. (2012). Correlation of Alzheimer Disease Neuropathologic Changes with Cognitive Status: A Review of the Literature. J. Neuropathol. Exp. Neurol..

[117] David A.B., Julie A.S., Robert S.W., Julia L.B., Steven E.A. (2004). Neurofibrillary Tangles Mediate the Association of Amyloid Load with Clinical Alzheimer Disease and Level of Cognitive Function. Arch. Neurol..

[118] Braak H., Braak E. (1991). Neuropathological Stageing of Alzheimer-Related Changes. Acta Neuropathol..

[119] Malpetti M., Kievit R.A., Passamonti L., Jones P.S., Tsvetanov K.A., Rittman T., Mak E., Nicastro N., Bevan-Jones W.R., Su L. (2020). Microglial Activation and Tau Burden Predict Cognitive Decline in Alzheimer’s Disease. Brain.

[120] Simoes S., Neufeld J.L., Triana-Baltzer G., Moughadam S., Chen E.I., Kothiya M., Qureshi Y.H., Patel V., Honig L.S., Kolb H. (2020). Tau and Other Protein Found in Alzheimer’s Disease Spinal Fluid Are Linked to Retromer-mediated Endosomal Traffic in Mice and Humans. Sci. Trans. Med..

[121] Pizzino G., Irrera N., Cucinotta M., Pallio G., Mannino F., Arcoraci V., Squadrito F., Altavilla D., Bitto A. (2017). Oxidative Stress: Harms and Benefits for Human Health. Oxid. Med. Cell. Longev..

[122] Lee K.H., Cha M., Lee B.H. (2020). Neuroprotective Effect of Antioxidants in the Brain. Int. J. Mol. Sci..

[123] Chen X., Guo C., Kong J. (2012). Oxidative Stress in Neurodegenerative Diseases. Neural Regen. Res..

[124] Huang W.J., Zhang X., Chen W.W. (2016). Role of Oxidative Stress in Alzheimer’s Disease (Review). Biomed. Rep..

[125] Sonnen J.A., Breitner J.C., Lovell M.A., Markesbery W.R., Quinn J.F., Montine T.J. (2008). Free Radical-Mediated Damage to Brain in Alzheimer’s Disease and Its Transgenic Mouse Models. Free Radic. Biol. Med..

[126] Markesbery W.R. (1997). Oxidative Stress Hypothesis in Alzheimer’s Disease. Free Radic. Biol. Med..

[127] Chun H., Im H., Kang Y.J., Kim Y., Shin J.H., Won W., Lim J., Ju Y., Park Y.M., Kim S. (2020). Severe Reactive Astrocytes Precipitate Pathological Hallmarks of Alzheimer’s Disease via H_2_O_2_^−^ Production. Nat. Neurosci..

[128] Mastroeni D., Khdour O.M., Delvaux E., Nolz J., Olsen G., Berchtold N., Cotman C., Hecht S.M., Coleman P.D. (2017). Nuclear but Not Mitochondrial-Encoded Oxidative Phosphorylation Genes Are Altered in Aging, Mild Cognitive Impairment, and Alzheimer’s Disease. Alzheimer’s Dement..

[129] Baldeiras I., Santana I., Proença M.T., Garrucho M.H., Pascoal R., Rodrigues A., Duro D., Oliveira C.R. (2008). Peripheral Oxidative Damage in Mild Cognitive Impairment and Mild Alzheimer’s Disease. J. Alzheimer’s Dis..

[130] Youssef P., Chami B., Lim J., Middleton T., Sutherland G.T., Witting P.K. (2018). Evidence Supporting Oxidative Stress in a Moderately Affected Area of the Brain in Alzheimer’s Disease. Sci. Rep..

[131] Craddock T.J.A., Tuszynski J.A., Chopra D., Casey N., Goldstein L.E., Hameroff S.R., Tanzi R.E. (2012). The Zinc Dyshomeostasis Hypothesis of Alzheimer’s Disease. PLoS ONE.

[132] Darshpreet K., Vivek S., Rahul D. (2019). Activation of Microglia and Astrocytes: A Roadway to Neuroinflammation and Alzheimer’s Disease. Inflammopharmacology.

[133] Galloway D.A., Phillips A.E.M., Owen D.R.J., Moore C.S. (2019). Phagocytosis in the Brain: Homeostasis and Disease. Front. Immunol..

[134] Weldon D.T., Rogers S.D., Ghilardi J.R., Finke M.P., Cleary J.P., O’Hare E., Esler W.P., Maggio J.E., Mantyh P.W. (1998). Fibrillar β-Amyloid Induces Microglial Phagocytosis, Expression of Inducible Nitric Oxide Synthase, and Loss of a Select Population of Neurons in the Rat CNS in Vivo. J. Neurosci..

[135] Amor S., Puentes F., Baker D., Van Der Valk P. (2010). Inflammation in Neurodegenerative Diseases. Immunology.

[136] Sarlus H., Heneka M.T. (2017). Microglia in Alzheimer’s Disease. J. Clin. Invest..

[137] Koenigsknecht J., Landreth G. (2004). Microglial Phagocytosis of Fibrillar β-Amyloid through a Β1 Integrin-Dependent Mechanism. J. Neurosci..

[138] Sheppard O., Coleman M.P., Durrant C.S. (2019). Lipopolysaccharide-Induced Neuroinflammation Induces Presynaptic Disruption through a Direct Action on Brain Tissue Involving Microglia-Derived Interleukin 1 Beta. J. Neuroinflamm..

[139] Sheng J.G., Mrak R.E., Griffin W.S.T. (1997). Glial-Neuronal Interactions in Alzheimer Disease: Progressive Association of IL^−^1α^+^ Microglia and S100β^+^ Astrocytes with Neurofibrillary Tangle Stages. J. Neuropathol. Exp. Neurol..

[140] Wang W.Y., Tan M.S., Yu J.T., Tan L. (2015). Role of Pro-Inflammatory Cytokines Released from Microglia in Alzheimer’s Disease. Ann. Transl. Med..

[141] Brosseron F., Krauthausen M., Kummer M., Heneka M.T. (2014). Body Fluid Cytokine Levels in Mild Cognitive Impairment and Alzheimer’s Disease: A Comparative Overview. Mol. Neurobiol..

[142] Wood H. (2017). Alzheimer Disease: Twin Peaks of Microglial Activation Observed in Alzheimer Disease. Nat. Rev. Neurol..

[143] Sanchez J.R., Marsh S., McIntyre L., Davtyan H., Walsh C., Blurton-Jones M. (2020). Cytotoxic T Cells Infiltrate the Brain and Interact with Microglia to Reduce Alzheimer’s Disease Pathogenesis. J. Immunol..

[144] Fisher Y., Nemirovsky A., Baron R., Monsonego A. (2010). T Cells Specifically Targeted to Amyloid Plaques Enhance Plaque Clearance in a Mouse Model of Alzheimer’s Disease. PLoS ONE.

[145] Togo T., Akiyama H., Iseki E., Kondo H., Ikeda K., Kato M., Oda T., Tsuchiya K., Kosaka K. (2002). Occurrence of T Cells in the Brain of Alzheimer’s Disease and Other Neurological Diseases. J. Neuroimmunol..

[146] Bulati M., Martorana A., Gervasi F., Camarda C., Azzarello D.M., Monastero R., Caruso C., Colonna-Romano G. (2015). Double Negative (IgG^+^IgD^−^CD27^−^) B Cells Are Increased in a Cohort of Moderate-Severe Alzheimer’s Disease Patients and Show a pro-Inflammatory Trafficking Receptor Phenotype. J. Alzheimer’s Dis..

[147] Söllvander S., Ekholm-Pettersson F., Brundin R.M., Westman G., Kilander L., Paulie S., Lannfelt L., Sehlin D. (2015). Increased Number of Plasma B Cells Producing Autoantibodies against Aβ 42 Protofibrils in Alzheimer’s Disease. J. Alzheimer’s Dis..

[148] Dionisio-Santos D.A., Olschowka J.A., O’Banion M.K. (2019). Exploiting Microglial and Peripheral Immune Cell Crosstalk to Treat Alzheimer’s Disease. J. Neuroinflamm..

[149] Panza F., Lozupone M., Solfrizzi V., Sardone R., Piccininni C., Dibello V., Stallone R., Giannelli G., Bellomo A., Greco A. (2018). BACE Inhibitors in Clinical Development for the Treatment of Alzheimer’s Disease. Expert Rev. Neurother..

[150] Kennedy M.E., Stamford A.W., Chen X., Cox K., Cumming J.N., Dockendorf M.F., Egan M., Ereshefsky L., Hodgson R.A., Hyde L.A. (2016). The BACE1 Inhibitor Verubecestat (MK-8931) Reduces CNS b-Amyloid in Animal Models and in Alzheimer’s Disease Patients. Sci. Transl. Med..

[151] Cebers G., Lejeune T., Attalla B., Soderberg M., Alexander R.C., Haeberlein S.B., Kugler A.R., Ingersoll E.W., Platz S., Scott C.W. (2016). Reversible and Species-Specific Depigmentation Effects of AZD3293, a BACE Inhibitor for the Treatment of Alzheimer’s Disease, Are Related to BACE2 Inhibition and Confined to Epidermis and Hair. J. Prev. Alzheimer’s Dis..

[152] US National Library of Medicine (2019). A 24-Month Study to Evaluate the Efficacy and Safety of Elenbecestat (E2609) in Subjects with Early Alzheimer’s Disease (MissionAD1). https://clinicaltrials.gov/ct2/show/NCT03036280.

[153] Janssen Research and Development An Efficacy and Safety Study of Atabecestat in Participants Who Are Asymptomatic at Risk for Developing Alzheimer’s Dementia. https://clinicaltrials.gov/ct2/show/NCT02569398.

[154] Novartis Pharmaceuticals, Banner Alzheimer’s Institute (2020). A Study of CNP520 Versus Placebo in Participants at Risk for the Onset of Clinical Symptoms of Alzheimer’s Disease. https://clinicaltrials.gov/ct2/show/NCT03131453.

[155] Vladimir C., Stephen S., Christopher H.V.D., Bruno D., Niels A., Mark B., Craig C., Hilkka S., Stephen T., Thomas S. (2015). Targeting Prodromal Alzheimer Disease with Avagacestat: A Randomized Clinical Trial. JAMA Neurol..

[156] Tanokashira D., Mamada N., Yamamoto F., Taniguchi K., Tamaoka A., Lakshmana M.K., Araki W. (2017). The Neurotoxicity of Amyloid β-Protein Oligomers Is Reversible in a Primary Neuron Model. Mol. Brain.

[157] Montoliu-Gaya L., Villegas S. (2016). Aβ-Immunotherapeutic Strategies: A Wide Range of Approaches for Alzheimer’s Disease Treatment. Expert Rev. Mol. Med..

[158] Sevigny J., Chiao P., Bussière T., Weinreb P.H., Williams L., Maier M., Dunstan R., Salloway S., Chen T., Ling Y. (2016). The Antibody Aducanumab Reduces Aβ Plaques in Alzheimer’s Disease. Nature.

[159] Loeffler D.A. (2013). Intravenous Immunoglobulin and Alzheimer’s Disease: What Now?. J. Neuroinflamm..

[160] Price K.A., Crouch P.J., White A.R. (2007). Therapeutic Treatment of Alzheimer’s Disease Using Metal Complexing Agents. Recent Pat. CNS Drug Discov..

[161] Ritchie C.W., Bush A.I., Mackinnon A., Macfarlane S., Mastwyk M., MacGregor L., Kiers L., Cherny R., Li Q.X., Tammer A. (2003). Metal-Protein Attenuation with Iodochlorhydroxyquin (Clioquinol) Targeting Aβ Amyloid Deposition and Toxicity in Alzheimer Disease: A Pilot Phase 2 Clinical Trial. Arch. Neurol..

[162] Arbiser J.L., Kraeft S.K., Van Leeuwen R., Hurwitz S.J., Selig M., Dickersin G.R., Flint A., Byers H.R., Chen L.B. (1998). Clioquinol-Zinc Chelate: A Candidate Causative Agent of Subacute Myelo-Optic Neuropathy. Mol. Med..

[163] Makin S. (2018). The Amyloid Hypothesis on Trial. Nature.

[164] Lansdall C.J. (2014). An Effective Treatment for Alzheimer’s Disease Must Consider Both Amyloid and Tau. Biosci. Horiz..

[165] Panza F., Lozupone M., Logroscino G., Imbimbo B.P. (2019). A Critical Appraisal of Amyloid-β-Targeting Therapies for Alzheimer Disease. Nat. Rev. Neurol..

[166] Piette F., Belmin J., Vincent H., Schmidt N., Pariel S., Verny M., Marquis C., Mely J., Hugonot-Diener L., Kinet J.P. (2011). Masitinib as an Adjunct Therapy for Mild-to-Moderate Alzheimer’s Disease: A Randomised, Placebo-Controlled Phase 2 Trial. Alzheimer’s Res. Ther..

[167] Congdon E.E., Sigurdsson E.M. (2018). Tau-Targeting Therapies for Alzheimer Disease. Nat. Rev. Neurol..

[168] Augustinack J.C., Sanders J.L., Tsai L.H., Hyman B.T. (2002). Colocalization and Fluorescence Resonance Energy Transfer between Cdk5 and AT8 Suggests a Close Association in Pre-Neurofibrillary Tangles and Neurofibrillary Tangles. J. Neuropathol. Exp. Neurol..

[169] Sengupta A., Novak M., Grundke-Iqbal I., Iqbal K. (2006). Regulation of Phosphorylation of Tau by Cyclin-Dependent Kinase 5 and Glycogen Synthase Kinase-3 at Substrate Level. FEBS Lett..

[170] Caccamo A., Oddo S., Tran L.X., LaFerla F.M. (2007). Lithium Reduces Tau Phosphorylation but Not Aβ or Working Memory Deficits in a Transgenic Model with Both Plaques and Tangles. Am. J. Pathol..

[171] Lee J.K., Kim N.J. (2017). Recent Advances in the Inhibition of P38 MAPK as a Potential Strategy for the Treatment of Alzheimer’s Disease. Molecules.

[172] Yadikar H., Torres I., Aiello G., Kurup M., Yang Z., Lin F., Kobeissy F., Yost R., Wang K. (2020). Screening of Tau Protein Kinase Inhibitors in a Tauopathy-Relevant Cell-Based Model of Tau Hyperphosphorylation and Oligomerization. PLoS ONE.

[173] Sontag J.M., Sontag E. (2014). Protein Phosphatase 2A Dysfunction in Alzheimer’s Disease. Front. Mol. Neurosci..

[174] Millward T.A., Zolnierowicz S., Hemmings B.A. (1999). Regulation of Protein Kinase Cascades by Protein Phosphatase 2A. Trends Biochem. Sci..

[175] Gong C.-X., Shaikh S., Wang J.-Z., Zaidi T., Grundke-Iqbal I., Iqbal K. (1995). Phosphatase Activity Toward Abnormally Phosphorylated τ: Decrease in Alzheimer Disease Brain. J. Neurochem..

[176] Braithwaite S.P., Stock J.B., Lombroso P.J., Nairn A.C. (2012). Protein Phosphatases and Alzheimer’s Disease. Prog. Mol. Biol. Transl. Sci..

[177] Liu F., Grundke-Iqbal I., Iqbal K., Gong C.X. (2005). Contributions of Protein Phosphatases PP1, PP2A, PP2B and PP5 to the Regulation of Tau Phosphorylation. Eur. J. Neurosci..

[178] McKenzie-Nickson S., Chan J., Perez K., Hung L.W., Cheng L., Sedjahtera A., Gunawan L., Adlard P.A., Hayne D.J., McInnes L.E. (2018). Modulating Protein Phosphatase 2A Rescues Disease Phenotype in Neurodegenerative Tauopathies. ACS Chem. Neurosci..

[179] Morita K., He S., Nowak R.P., Wang J., Zimmerman M.W., Fu C., Durbin A.D., Martel M.W., Prutsch N., Gray N.S. (2020). Allosteric Activators of Protein Phosphatase 2A Display Broad Antitumor Activity Mediated by Dephosphorylation of MYBL2. Cell.

[180] Iqbal K., Liu F., Gong C.-X., Grundke-Iqbal I. (2010). Tau in Alzheimer Disease and Related Tauopathies. Curr. Alzheimer Res..

[181] Khanna M.R., Kovalevich J., Lee V.M.Y., Trojanowski J.Q., Brunden K.R. (2016). Therapeutic Strategies for the Treatment of Tauopathies: Hopes and Challenges. Alzheimer’s Dement..

[182] Bulic B., Pickhardt M., Schmidt B., Mandelkow E.M., Waldmann H., Mandelkow E. (2009). Development of Tau Aggregation Inhibitors for Alzheimer’s Disease. Angew. Chem..

[183] Akoury E., Pickhardt M., Gajda M., Biernat J., Mandelkow E., Zweckstetter M. (2013). Mechanistic Basis of Phenothiazine-Driven Inhibition of Tau Aggregation. Angew. Chem..

[184] Jadhav S., Avila J., Schöll M., Kovacs G.G., Kövari E., Skrabana R., Evans L.D., Kontsekova E., Malawska B., de Silva R. (2019). A Walk through Tau Therapeutic Strategies. Acta Neuropathol. Commun..

[185] Zheng Q., Kebede M.T., Kemeh M.M., Islam S., Lee B., Bleck S.D., Wurfl L.A., Lazo N.D. (2019). Inhibition of the Self-Assembly of Aβ and of Tau by Polyphenols: Mechanistic Studies. Molecules.

[186] van Bebber F., Paquet D., Hruscha A., Schmid B., Haass C. (2010). Methylene Blue Fails to Inhibit Tau and Polyglutamine Protein Dependent Toxicity in Zebrafish. Neurobiol. Dis..

[187] Saraiva C., Praça C., Ferreira R., Santos T., Ferreira L., Bernardino L. (2016). Nanoparticle-Mediated Brain Drug Delivery: Overcoming Blood-Brain Barrier to Treat Neurodegenerative Diseases. J. Control. Release.

[188] Taniguchi S., Suzuki N., Masuda M., Hisanaga S.I., Iwatsubo T., Goedert M., Hasegawa M. (2004). Inhibition of Heparin-Induced Tau Filament Formation by Phenothiazines, Polyphenols, and Porphyrins. J. Biol. Chem..

[189] Perez-Nievas B.G., Serrano-Pozo A. (2018). Deciphering the Astrocyte Reaction in Alzheimer’s Disease. Front. Aging Neurosci..

[190] Sriram K., Benkovic S.A., Hebert M.A., Miller D.B., O’Callaghan J.P. (2004). Induction of Gp130-Related Cytokines and Activation of JAK2/STAT3 Pathway in Astrocytes Precedes up-Regulation of Glial Fibrillary Acidic Protein in the 1-Methyl-4-Phenyl-1,2,3,6-Tetrahydropyridine Model of Neurodegeneration: Key Signaling Pathway for Ast. J. Biol. Chem..

[191] Yang X., He G., Hao Y., Chen C., Li M., Wang Y., Zhang G., Yu Z. (2010). The Role of the JAK2-STAT3 Pathway in pro-Inflammatory Responses of EMF-Stimulated N9 Microglial Cells. J. Neuroinflamm..

[192] Ceyzériat K., Ben Haim L., Denizot A., Pommier D., Matos M., Guillemaud O., Palomares M.A., Abjean L., Petit F., Gipchtein P. (2018). Modulation of Astrocyte Reactivity Improves Functional Deficits in Mouse Models of Alzheimer’s Disease. Acta Neuropathol. Commun..

[193] Reichenbach N., Delekate A., Plescher M., Schmitt F., Krauss S., Blank N., Halle A., Petzold G.C. (2019). Inhibition of Stat3-mediated Astrogliosis Ameliorates Pathology in an Alzheimer’s Disease Model. EMBO Mol. Med..

[194] Yang Y., Wang H., Kouadir M., Song H., Shi F. (2019). Recent Advances in the Mechanisms of NLRP3 Inflammasome Activation and Its Inhibitors. Cell Death Dis..

[195] Heneka M.T., Kummer M.P., Stutz A., Delekate A., Schwartz S., Vieira-Saecker A., Griep A., Axt D., Remus A., Tzeng T.C. (2013). NLRP3 Is Activated in Alzheimer’s Disease and Contributes to Pathology in APP/PS1 Mice. Nature.

[196] Dugan L.L., Ali S.S., Shekhtman G., Roberts A.J., Lucero J., Quick K.L., Behrens M.M. (2009). IL-6 Mediated Degeneration of Forebrain GABAergic Interneurons and Cognitive Impairment in Aged Mice through Activation of Neuronal NADPH Oxidase. PLoS ONE.

[197] Valerio A., Boroni F., Benarese M., Sarnico I., Ghisi V., Bresciani L.G., Ferrario M., Borsani G., Spano P., Pizzi M. (2006). NF-ΚB Pathway: A Target for Preventing β-Amyloid (Aβ)-Induced Neuronal Damage and Aβ42 Production. Eur. J. Neurosci..

[198] Bales K.R., Du Y., Dodel R.C., Yan G.M., Hamilton-Byrd E., Paul S.M. (1998). The NF-ΚB/Rel Family of Proteins Mediates A β-Induced Neurotoxicity and Glial Activation. Mol. Brain Res..

[199] Liu T., Zhang L., Joo D., Sun S.C. (2017). NF-ΚB Signaling in Inflammation. Signal Transduct. Target. Ther..

[200] Zhang J., Shen B., Lin A. (2007). Novel Strategies for Inhibition of the P38 MAPK Pathway. Trends Pharmacol. Sci..

[201] Coulthard L.R., White D.E., Jones D.L., McDermott M.F., Burchill S.A. (2009). P38MAPK: Stress Responses from Molecular Mechanisms to Therapeutics. Trends Mol. Med..

[202] Kheiri G., Dolatshahi M., Rahmani F., Rezaei N. (2018). Role of P38/MAPKs in Alzheimer’s Disease: Implications for Amyloid Beta Toxicity Targeted Therapy. Rev. Neurosci..

[203] Gee M.S., Son S.H., Jeon S.H., Do J., Kim N., Ju Y.J., Lee S.J., Chung E.K., Inn K.S., Kim N.J. (2020). A Selective P38α/β MAPK Inhibitor Alleviates Neuropathology and Cognitive Impairment, and Modulates Microglia Function in 5XFAD Mouse. Alzheimer’s Res. Ther..

[204] Du Y., Du Y., Zhang Y., Huang Z., Fu M., Li J., Pang Y., Lei P., Wang Y.T., Song W. (2019). MKP-1 Reduces Aβ Generation and Alleviates Cognitive Impairments in Alzheimer’s Disease Models. Signal Transduct. Target. Ther..

[205] Sompol P., Norris C.M. (2018). Ca^2+^, Astrocyte Activation and Calcineurin/NFAT Signaling in Age-Related Neurodegenerative Diseases. Front. Aging Neurosci..

[206] Nagamoto-Combs K., Combs C.K. (2010). Microglial Phenotype Is Regulated by Activity of the Transcription Factor, NFAT (Nuclear Factor of Activated T Cells). J. Neurosci..

[207] Furman J.L., Sama D.M., Gant J.C., Beckett T.L., Murphy M.P., Bachstetter A.D., van Eldik L.J., Norris C.M. (2012). Targeting Astrocytes Ameliorates Neurologic Changes in a Mouse Model of Alzheimer’s Disease. J. Neurosci..

[208] Taglialatela G., Rastellini C., Cicalese L. (2015). Reduced Incidence of Dementia in Solid Organ Transplant Patients Treated with Calcineurin Inhibitors. J. Alzheimer’s Dis..

[209] Zhao Y., Wu X., Li X., Jiang L.L., Gui X., Liu Y., Sun Y., Zhu B., Piña-Crespo J.C., Zhang M. (2018). TREM2 Is a Receptor for β-Amyloid That Mediates Microglial Function. Neuron.

[210] Jiang T., Tan L., Zhu X.C., Zhou J.S., Cao L., Tan M.S., Wang H.F., Chen Q., Zhang Y.D., Yu J.T. (2015). Silencing of TREM2 Exacerbates Tau Pathology, Neurodegenerative Changes, and Spatial Learning Deficits in P301S Tau Transgenic Mice. Neurobiol. Aging.

[211] Jiang T., Zhang Y.D., Chen Q., Gao Q., Zhu X.C., Zhou J.S., Shi J.Q., Lu H., Tan L., Yu J.T. (2016). TREM2 Modifies Microglial Phenotype and Provides Neuroprotection in P301S Tau Transgenic Mice. Neuropharmacology.

[212] Bisht K., Sharma K.P., Lecours C., Sánchez M.G., El Hajj H., Milior G., Olmos-Alonso A., Gómez-Nicola D., Luheshi G., Vallières L. (2016). Dark Microglia: A New Phenotype Predominantly Associated with Pathological States. Glia.

[213] Fitz N.F., Wolfe C.M., Playso B.E., Biedrzycki R.J., Lu Y., Nam K.N., Lefterov I., Koldamova R. (2020). Trem2 Deficiency Differentially Affects Phenotype and Transcriptome of Human APOE3 and APOE4 Mice. Mol. Neurodegener..

[214] Yeh F.L., Hansen D.V., Sheng M. (2017). TREM2, Microglia, and Neurodegenerative Diseases. Trends Mol. Med..

[215] Korvatska O., Leverenz J.B., Jayadev S., McMillan P., Kurtz I., Guo X., Rumbaugh M., Matsushita M., Girirajan S., Dorschner M.O. (2015). R47H Variant of TREM2 Associated With Alzheimer Disease in a Large Late-Onset Family. JAMA Neurol..

[216] Ren S., Yao W., Tambini M.D., Yin T., Norris K.A., D’Adamio L. (2020). Microglia TREM2R47H Alzheimer-Linked Variant Enhances Excitatory Transmission and Reduces LTP via Increased TNF-α Levels. eLife.

[217] Guerreiro R., Wojtas A., Bras J., Carrasquillo M., Rogaeva E., Majounie E., Cruchaga C., Sassi C., Kauwe J.S.K., Younkin S. (2013). TREM2 Variants in Alzheimer’s Disease. N. Engl. J. Med..

[218] Jiang T., Tan L., Zhu X.C., Zhang Q.Q., Cao L., Tan M.S., Gu L.Z., Wang H.F., Ding Z.Z., Zhang Y.D. (2014). Upregulation of TREM2 Ameliorates Neuropathology and Rescues Spatial Cognitive Impairment in a Transgenic Mouse Model of Alzheimer’s Disease. Neuropsychopharmacology.

[219] Zhong L., Xu Y., Zhuo R., Wang T., Wang K., Huang R., Wang D., Gao Y., Zhu Y., Sheng X. (2019). Soluble TREM2 Ameliorates Pathological Phenotypes by Modulating Microglial Functions in an Alzheimer’s Disease Model. Nat. Commun..

[220] Lee C.Y.D., Daggett A., Gu X., Jiang L., Langfelder P., Li X., Wang N., Zhao Y., Park C.S., Cooper Y. (2018). Elevated TREM2 Gene Dosage Reprograms Microglia Responsivity and Ameliorates Pathological Phenotypes in Alzheimer’s Disease Models. Neuron.

[221] Kielian T. (2006). Toll-like Receptors in Central Nervous System Glial Inflammation and Homeostasis. J. Neurosci. Res..

[222] Lin C., Zhao S., Zhu Y., Fan Z., Wang J., Zhang B., Chen Y. (2019). Microbiota-Gut-Brain Axis and Toll-like Receptors in Alzheimer’s Disease. Comput. Struct. Biotechnol. J..

[223] Chen K., Iribarren P., Hu J., Chen J., Gong W., Cho E.H., Lockett S., Dunlop N.M., Ji M.W. (2006). Activation of Toll-like Receptor 2 on Microglia Promotes Cell Uptake of Alzheimer Disease-Associated Amyloid β Peptide. J. Biol. Chem..

[224] McDonald C.L., Hennessy E., Rubio-Araiz A., Keogh B., McCormack W., McGuirk P., Reilly M., Lynch M.A. (2016). Inhibiting TLR2 Activation Attenuates Amyloid Accumulation and Glial Activation in a Mouse Model of Alzheimer’s Disease. Brain Behav. Immun..

[225] Kazuki T., Hong-Duck K., Jing-Ji J., Adam M.J., Ling L., Ken-Ichiro F. (2008). Role of Toll-like Receptor Signalling in Aβ Uptake and Clearance. Brain.

[226] Hughes C., Choi M.L., Yi J.H., Kim S.C., Drews A., George-Hyslop P.S., Bryant C., Gandhi S., Cho K., Klenerman D. (2020). Beta Amyloid Aggregates Induce Sensitised TLR4 Signalling Causing Long-Term Potentiation Deficit and Rat Neuronal Cell Death. Commun. Biol..

[227] Deane R.J. (2012). Is RAGE Still a Therapeutic Target for Alzheimers Disease?. Future Med. Chem..

[228] Fang F., Lue L.-F., Yan S., Xu H., Luddy J.S., Chen D., Walker D.G., Stern D.M., Yan S., Schmidt A.M. (2010). RAGE-dependent Signaling in Microglia Contributes to Neuroinflammation, Aβ Accumulation, and Impaired Learning/Memory in a Mouse Model of Alzheimer’s Disease. FASEB J..

[229] Baek H., Ye M., Kang G., Lee C., Lee G., Choi B., Jung J., Kim H., Lee S., Kim J.S. (2016). Neuroprotective Effects of CD4^+^ CD25^+^ Foxp3^+^ Regulatory T Cells in a 3xTg-AD Alzheimer’s Disease Model. Oncotarget.

[230] Dansokho C., Ahmed D.A., Aid S., Toly-Ndour C., Chaigneau T., Calle V., Cagnard N., Holzenberger M., Piaggio E., Aucouturier P. (2016). Regulatory T Cells Delay Disease Progression in Alzheimer-like Pathology. Brain.

[231] Engelhardt B. (2010). T Cell Migration into the Central Nervous System during Health and Disease: Different Molecular Keys Allow Access to Different Central Nervous System Compartments. Clin. Exp. Neuroimmunol..

[232] Fu H.J., Liu B., Frost J.L., Lemere C.A. (2010). Amyloid-β Immunotherapy for Alzheimers Disease. CNS Neurol. Disord. Drug Targets.

[233] Monsonego A., Imitola J., Zota V., Oida T., Weiner H.L. (2004). Microglia-Mediated Nitric Oxide Cytotoxicity of T Cells Following Amyloid β Peptide Presentation to Th1 Cells. J. Immunol..

[234] Kim S.H., Noh M.Y., Kim H.-J., Oh K.-W., Park J., Lee S., Moon Y., Kim Y.-E., Bae J.S., Jin H.K. (2019). A Therapeutic Strategy for Alzheimer’s Disease Focused on Immune-Inflammatory Modulation. Dement. Neurocogn. Disord..

[235] NIH (2018). Safety and Efficacy Study of ALZT-OP1 in Subjects with Evidence of Early Alzheimer’s Disease. https://clinicaltrials.gov/ct2/show/NCT02547818.

[236] Van Eldik L.J., Carrillo M.C., Cole P.E., Feuerbach D., Greenberg B.D., Hendrix J.A., Kennedy M., Kozauer N., Margolin R.A., Molinuevo J.L. (2016). The Roles of Inflammation and Immune Mechanisms in Alzheimers Disease. Alzheimer’s Dement. Transl. Res. Clin. Interv..

[237] Allen S.J., Watson J.J., Dawbarn D. (2011). The Neurotrophins and Their Role in Alzheimers Disease. Curr. Neuropharmacol..

[238] Hampel H., Caraci F., Cuello A.C., Caruso G., Nisticò R., Corbo M., Baldacci F., Toschi N., Garaci F., Chiesa P.A. (2020). A Path Toward Precision Medicine for Neuroinflammatory Mechanisms in Alzheimer’s Disease. Front. Immunol..

[239] Shohayeb B., Diab M., Ahmed M., Ng D.C.H. (2018). Factors That Influence Adult Neurogenesis as Potential Therapy. Transl. Neurodegener..

[240] Weissmiller A.M., Wu C. (2012). Current Advances in Using Neurotrophic Factors to Treat Neurodegenerative Disorders. Transl. Neurodegener..

[241] Laske C., Stransky E., Leyhe T., Eschweiler G.W., Wittorf A., Richartz E., Bartels M., Buchkremer G., Schott K. (2006). Stage-Dependent BDNF Serum Concentrations in Alzheimer’s Disease. J. Neural Transm..

[242] Alicia M., Jordan S., Maria-Jose M., Reginald M.H., Janet K., Judi M.W., John E.L. (2018). The Effect of Dietary Supplementation on Brain-Derived Neurotrophic Factor and Cognitive Functioning in Alzheimer’s Dementia. J. Clin. Transl. Res..

[243] Jönhagen M.E., Nordberg A., Amberla K., Bäckman L., Ebendal T., Meyerson B., Olson L., Seiger Å., Shigeta M., Theodorsson E. (1998). Intracerebroventricular Infusion of Nerve Growth Factor in Three Patients with Alzheimer’s Disease. Dement. Geriatr. Cogn. Disord..

[244] Yaar M., Zhai S., Panova I., Fine R.E., Eisenhauer P.B., Blusztajn J.K. (2007). A Cyclic Peptide That Binds P75^NTR^ Protects Neurones from Beta Amyloid (1–40)-Induced Cell Death. Neuropathol. Appl. Neurobiol..

[245] Rafii M.S., Baumann T.L., Bakay R.A.E., Ostrove J.M., Siffert J., Fleisher A.S., Herzog C.D., Barba D., Pay M., Salmon D.P. (2014). A Phase1 Study of Stereotactic Gene Delivery of AAV2-NGF for Alzheimer’s Disease. Alzheimer’s Dement..

[246] Skaper S.D. (2011). Peptide Mimetics of Neurotrophins and Their Receptors. Curr. Pharm. Des..

[247] Liu X., Chan C., Jang S., Pradoldej S., Huang J., He K., Phun L.H. (2010). A Synthetic 7,8-Dihydroxyflavone Derivative Promotes Neurogenesis and Exhibits Potent Antidepressant Effect. J. Med. Chem..

[248] Augusto-Oliveira M., Arrifano G.P., Lopes-Araújo A., Santos-Sacramento L., Takeda P.Y., Anthony D.C., Malva J.O., Crespo-Lopez M.E. (2019). What Do Microglia Really Do in Healthy Adult Brain?. Cells.

[249] Thomsen G.M., Avalos P., Ma A.A., Alkaslasi M., Cho N., Wyss L., Vit J.P., Godoy M., Suezaki P., Shelest O. (2018). Transplantation of Neural Progenitor Cells Expressing Glial Cell Line-Derived Neurotrophic Factor into the Motor Cortex as a Strategy to Treat Amyotrophic Lateral Sclerosis. Stem Cells.

[250] Cardoso T., Adler A.F., Mattsson B., Hoban D.B., Nolbrant S., Wahlestedt J.N., Kirkeby A., Grealish S., Björklund A., Parmar M. (2018). Target-Specific Forebrain Projections and Appropriate Synaptic Inputs of HESC-Derived Dopamine Neurons Grafted to the Midbrain of Parkinsonian Rats. J. Comp. Neurol..

[251] Henriques D., Moreira R., Schwamborn J., de Almeida L.P., Mendonça L.S. (2019). Successes and Hurdles in Stem Cells Application and Production for Brain Transplantation. Front. Neurosci..

[252] Wu Z., Parry M., Hou X.Y., Liu M.H., Wang H., Cain R., Pei Z.F., Chen Y.C., Guo Z.Y., Abhijeet S. (2020). Gene Therapy Conversion of Striatal Astrocytes into GABAergic Neurons in Mouse Models of Huntington’s Disease. Nat. Commun..

[253] Ricci G., Volpi L., Pasquali L., Petrozzi L., Siciliano G. (2009). Astrocyte—Neuron Interactions in Neurological Disorders. J. Biol. Phys..

[254] Parpura V., Heneka M.T., Montana V., Oliet S.H.R., Schousboe A., Haydon P.G., Stout R.F., Spray D.C., Reichenbach A., Pannicke T. (2012). Glial Cells in (Patho)Physiology. J. Neurochem..

[255] Choi S.H., Tanzi R.E. (2019). Is Alzheimer’s Disease a Neurogenesis Disorder?. Cell Stem Cell.

[256] Lee J.K., Jin H.K., Endo S., Schuchman E.H., Carter J.E., Bae J.S. (2010). Intracerebral Transplantation of Bone Marrow-Derived Mesenchymal Stem Cells Reduces Amyloid-Beta Deposition and Rescues Memory Deficits in Alzheimer’s Disease Mice by Modulation of Immune Responses. Stem Cells.

[257] Ziyuan G., Lei Z., Zheng W., Yuchen C., Fan W., Gong C. (2014). In Vivo Direct Reprogramming of Reactive Glial Cells into Functional Neurons after Brain Injury and in an Alzheimer’s Disease Model. Cell Stem Cell.

[258] Chen Y.C., Ma N.X., Pei Z.F., Wu Z., Do-Monte F.H., Keefe S., Yellin E., Chen M.S., Yin J.C., Lee G. (2020). A NeuroD1 AAV-Based Gene Therapy for Functional Brain Repair after Ischemic Injury through In Vivo Astrocyte-to-Neuron Conversion. Mol. Ther..

[259] Han F., Bi J., Qiao L., Arancio O. (2020). Stem Cell Therapy for Alzheimer’s Disease. Adv. Exp. Med. Biol..

[260] Santamaria G., Brandi E., La Vitola P., Grandi F., Ferrara G., Pischiutta F., Vegliante G., Zanier E.R., Re F., Uccelli A. (2020). Intranasal Delivery of Mesenchymal Stem Cell Secretome Repairs the Brain of Alzheimer’s Mice. Cell Death Differ..

[261] Li H., Chen G. (2016). In Vivo Reprogramming for CNS Repair: Regenerating Neurons from Endogenous Glial Cells. Neuron.

[262] Das G., Gupta V., Ghosh S. (2019). Glial-Neuron Transformation by “Chemical Cocktail”. ACS Chem. Neurosci..

[263] Lunn J.S., Sakowski S.A., Hur J., Feldman E.L. (2011). Stem Cell Technology for Neurodegenerative Diseases. Ann. Neurol..

[264] Urrutia D.N., Caviedes P., Mardones R. (2019). Comparative Study of the Neural Differentiation Capacity of Mesenchymal Stromal Cells from Different Tissue Sources: An Approach for Their Use in Neural Regeneration Therapies. PLoS ONE.

[265] Matsushita T., Kibayashi T., Katayama T., Yamashita Y., Suzuki S., Kawamata J., Honmou O., Minami M., Shimohama S. (2011). Mesenchymal Stem Cells Transmigrate across Brain Microvascular Endothelial Cell Monolayers through Transiently Formed Inter-Endothelial Gaps. Neurosci. Lett..

[266] Kim S., Chang K.A., Kim J.A., Park H.G., Ra J.C., Kim H.S., Suh Y.H. (2012). The Preventive and Therapeutic Effects of Intravenous Human Adipose-Derived Stem Cells in Alzheimer’s Disease Mice. PLoS ONE.

[267] Hayashi Y., Lin H.T., Lee C.C., Tsai K.J. (2020). Effects of Neural Stem Cell Transplantation in Alzheimer’s Disease Models. J. Biomed. Sci..

[268] Ahmed A.I., Zaben M., Gray W.P. (2011). Stem Cells in the Adult Human Brain. Br. J. Neurosurg..

[269] Ager R.R., Davis J.L., Agazaryan A., Benavente F., Poon W.W., LaFerla F.M., Blurton-Jones M. (2015). Human Neural Stem Cells Improve Cognition and Promote Synaptic Growth in Two Complementary Transgenic Models of Alzheimer’s Disease and Neuronal Loss. Hippocampus.

[270] Nori S., Okada Y., Nishimura S., Sasaki T., Itakura G., Kobayashi Y., Renault-mihara F., Shimizu A., Koya I., Yoshida R. (2015). Long-Term Safety Issues of IPSC-Based Cell Therapy in a Spinal Cord Injury Model: Oncogenic Transformation with Epithelial-Mesenchymal Transition. Stem Cell Rep..

[271] Dlouhy B.J., Awe O., Rao R.C., Kirby P.A., Hitchon P.W. (2014). Autograft-Derived Spinal Cord Mass Following Olfactory Mucosal Cell Transplantation in a Spinal Cord Injury Patient. J. Neurosurg..

[272] Herberts C.A., Kwa M.S.G., Hermsen H.P.H. (2011). Risk Factors in the Development of Stem Cell Therapy. J. Transl. Med..

[273] Frank L.H., Richard M.R., Burkhard B. (2015). Immune Attack: The Role of Inflammation in Alzheimer Disease. Nat. Rev. Neurosci..

